# Super-assembly of ER-phagy receptor Atg40 induces local ER remodeling at contacts with forming autophagosomal membranes

**DOI:** 10.1038/s41467-020-17163-y

**Published:** 2020-07-03

**Authors:** Keisuke Mochida, Akinori Yamasaki, Kazuaki Matoba, Hiromi Kirisako, Nobuo N. Noda, Hitoshi Nakatogawa

**Affiliations:** 1 School of Life Science and Technology, Tokyo Institute of Technology, Yokohama, Japan; 2 Institute of Microbial Chemistry (BIKAKEN), Tokyo, Japan; 3 Present Address: Cell Biology Center, Institute of Innovative Research, Tokyo Institute of Technology, Yokohama, Japan

**Keywords:** Macroautophagy, Endoplasmic reticulum, X-ray crystallography

## Abstract

The endoplasmic reticulum (ER) is selectively degraded by autophagy (ER-phagy) through proteins called ER-phagy receptors. In *Saccharomyces cerevisiae*, Atg40 acts as an ER-phagy receptor to sequester ER fragments into autophagosomes by binding Atg8 on forming autophagosomal membranes. During ER-phagy, parts of the ER are morphologically rearranged, fragmented, and loaded into autophagosomes, but the mechanism remains poorly understood. Here we find that Atg40 molecules assemble in the ER membrane concurrently with autophagosome formation via multivalent interaction with Atg8. Atg8-mediated super-assembly of Atg40 generates highly-curved ER regions, depending on its reticulon-like domain, and supports packing of these regions into autophagosomes. Moreover, tight binding of Atg40 to Atg8 is achieved by a short helix C-terminal to the Atg8-family interacting motif, and this feature is also observed for mammalian ER-phagy receptors. Thus, this study significantly advances our understanding of the mechanisms of ER-phagy and also provides insights into organelle fragmentation in selective autophagy of other organelles.

## Abstract (web summary)

The ER is subject to autophagy (ER-phagy) for turnover, with Atg40 acting as a receptor to sequester ER with Atg8 in autophagosomes. Here, the authors show that Atg40 is clustered by interaction with Atg8 to generate local membrane curvature and promote autophagosome packing.

## Introduction

The endoplasmic reticulum (ER) is a large membrane-bound organelle extending from the nuclear envelope to the cell periphery which serves multiple functions, including biogenesis of proteins and lipids^1^. The ER consists of interconnected tubules and sheets that generally enclose a single lumenal space. Reticulons and DP1/Yop1 families (reticulon-like proteins) generate ER membrane curvature to shape and maintain these ER tubules and the edges of ER sheets through short hairpin transmembrane domains (reticulon-like domains)^2^.

Autophagy pathways, an intracellular bulk degradation system induced by various stresses including nutrient starvation^3,4^, play a pivotal role in degradation and turnover of the ER^5^. In macroautophagy (hereafter, autophagy), a cup-shaped membrane vesicle, the isolation membrane (or phagophore) appears in the cytoplasm, expands, and closes to form a double-membrane vesicle, the autophagosome. In this process, a wide range of cellular materials are sequestered into the autophagosome and transported to the lysosome (in mammalian cells) or vacuole (in yeast and plant cells) for degradation. Autophagy degrades random portions of the cytoplasm, but can also selectively degrade certain proteins and organelles^6^. Landmark proteins called autophagy receptors bind specific targets and interact with Atg8-family proteins on the isolation membrane through the Atg8-family-interacting motif (AIM) (also called the LC3-interacting region, LIR). This linkage between autophagy receptors and Atg8 allows isolation membranes to sequester degradation targets efficiently. Several receptors for selective autophagy of the ER (ER-phagy) exist in yeast and mammals^5^. In the budding yeast *Saccharomyces cerevisiae*, we discovered two ER-phagy receptors, Atg39 and Atg40, that are responsible for degradation of distinct ER subdomains under nitrogen starvation conditions^7^. Atg39 and Atg40 localize to the perinuclear ER (equivalent to the nuclear envelope in yeast) and the cytoplasmic/cortical ER, respectively, and trigger autophagic degradation of the corresponding ER subdomains.

The transmembrane domain of Atg40 and the reticulon-like domain of DP1/Yop1 are predicted to be structurally similar, although they share no obvious sequence homology^7^. In mammalian cells, six ER-phagy receptors, FAM134B, RTN3, SEC62, CCPG1, TEX264, and ATL3, have been identified^8–14^; FAM134B and RTN3 possess the reticulon-like domains. The common presence of this domain in both proteins suggests that it is important in ER-phagy, but the mechanistic details remain obscure.

In this study, we demonstrated that Atg40 assumes a membrane topology similar to reticulon-like proteins, and that its overexpression suppresses defects caused by the absence of reticulon-like proteins in yeast cells. In addition, Atg40, by binding Atg8, forms a super-molecular assembly in the ER region that contacts the isolation membrane. This Atg40 assembly generates membrane curvature, thereby increasing the local concentration of the ER membrane in a manner dependent on its reticulon-like domain. Moreover, structural and biochemical analyses revealed that Atg40 strongly binds to Atg8 via a short helix following the AIM, and that this mode of interaction is also observed for FAM134B, RTN3, and SEC62. Our results suggest that the strong interaction between ER-phagy receptors and Atg8 drives local remodeling of the ER, a process important for efficient packing of this reticular/tubular organelle into autophagosomes.

## Results

### Atg40 generates membrane curvature in the ER

**Fig. 1:**
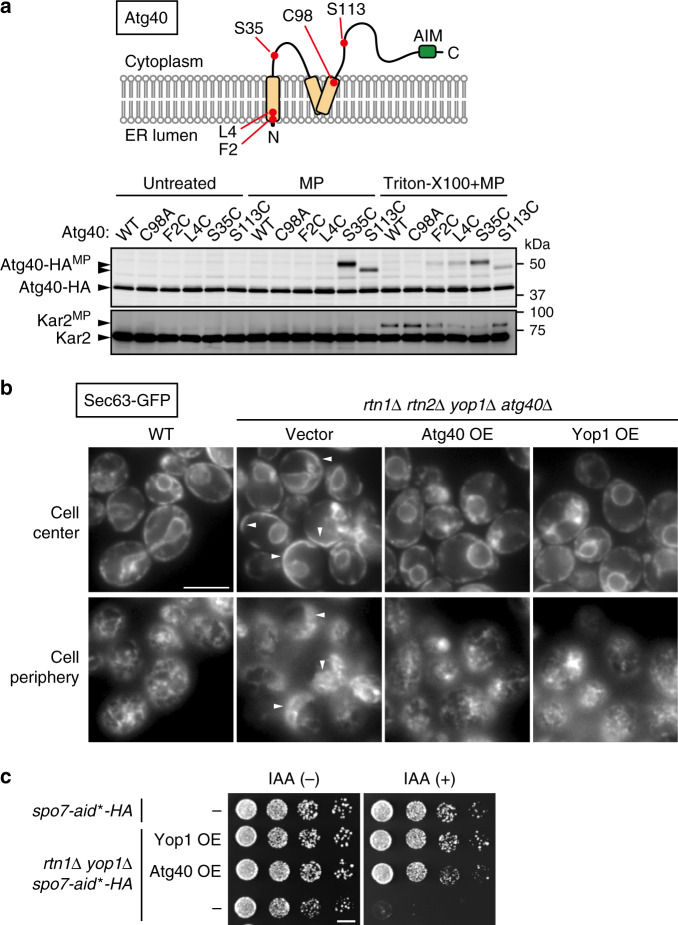
Atg40 is a reticulon-like protein. **a** Membrane fractions prepared from yeast cells expressing Atg40 mutants were treated with MP in the presence or absence of Triton X-100. The samples were analyzed by immunoblotting using antibodies against HA and Kar2. Atg40-HA^MP^, MP-modified Atg40-HA. The ER lumenal protein Kar2 served as a control. Kar2^MP^, MP-conjugated Kar2. **b** ER morphology was analyzed by fluorescence microscopy. Images were focused on the center or periphery of the cells. Arrowheads indicate abnormally expanded ER sheets. **c** spo7-aid*-HA cells were grown on YPD agar plates in the presence or absence of IAA. The experiments were repeated independently twice (**a**, **b**) or three times (**c**). Scale bars: 5 μm (**b**), 5 mm (**c**).

Structure-based sequence analysis suggested that Atg40 contains a reticulon-like domain^7^. To experimentally test this prediction, we performed a cysteine-modification assay using the membrane-impermeable, thiol-reactive reagent maleimide PEG (MP). In membrane fractions from cells, MP modifies cysteine residues exposed to the cytoplasm, but not those embedded in the membrane or organelle lumens. MP-modified proteins migrate more slowly than unmodified proteins in SDS-PAGE. Wild-type Atg40 tagged with the 6 × HA sequence contains only one cysteine residue (Cys98), but MP did not modify this protein or the Cys98-to-Ala (C98A) mutant, even when membranes were solubilized with Triton X-100 (Fig. 1a). Thus, the original Cys residue in Atg40 is somehow invulnerable to MP modification.

Next, we replaced four amino-acid residues of Atg40 individually with cysteine, yielding the F2C, L4C, S35C, and S113C mutants (Fig. 1a), which all exhibited normal ER-phagy (Supplementary Fig. 1a). MP modified all four mutants in the presence of Triton X-100, but only the S35C and S113C mutants in the absence of the detergent. These results suggest that the region between the two hydrophobic segments and the C-terminal region of Atg40, which contain Ser35 and Ser113, respectively, are exposed to the cytoplasm (Fig. 1a), implying the second hydrophobic segment of Atg40 adopts a hairpin-like structure in the ER membrane, similar to reticulon-like proteins.

Next, we investigated whether Atg40 can generate an ER region of high membrane curvature. As reported previously^2^, simultaneous deletion of the yeast reticulon-like proteins *RTN1*, *RTN2*, and *YOP1* caused abnormal expansion of ER sheets in cell periphery (Fig. 1b; Supplementary Fig. 1b). In the absence of these proteins, Atg40 still localized to highly curved ER regions (tubules and sheet edges) (Supplementary Fig. 1b). Although deletion of *ATG40* did not affect ER morphology (Supplementary Fig. 1c), Atg40 overexpression restored abnormal ER morphology in *rtn1*Δ *rtn2*Δ *yop1*Δ cells (Fig. 1b). Deletion of *SPO7*, which is involved in the regulation of phospholipid biosynthesis, is lethal in *rtn1*Δ *yop1*Δ cells^15,16^. We examined whether overexpression of Atg40 could rescue this lethality. To this end, Spo7 was conditionally knocked down using the auxin-inducible degron (AID) system^17^. Spo7 fused to the minimized AID (AID*) tag was degraded when cells were treated with the auxin indole-3-acetic acid (IAA) (Supplementary Fig. 1d)^18^. Although *rtn1*Δ *yop1*Δ spo7-aid*-HA cells had a growth defect in the presence of IAA, this defect was restored by overexpression of Atg40 (Fig. 1c), implying that overexpressed Atg40 can replace the function of these reticulon-like proteins. Taken together, we concluded that the ER-phagy receptor Atg40 is a bona fide reticulon-like protein that generates membrane curvature in the ER.

### The reticulon-like domain of Atg40 is crucial for ER-phagy

**Fig. 2:**
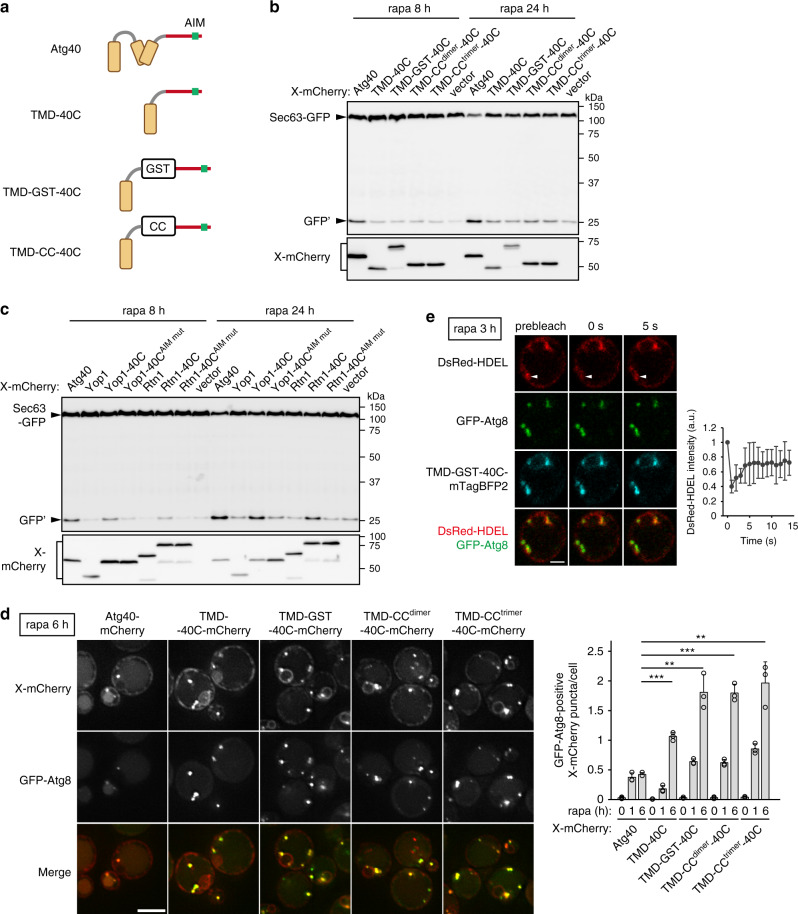
The reticulon-like domain of Atg40 is important for ER-phagy. **a** Schematic diagrams of fusion proteins containing the transmembrane domain (TMD) of Sec71 (24–58) and the C-terminal region (194–256) of Atg40 (40 C). CC, coiled-coil domain of Gcn4. **b**, **c** *SEC63-GFP atg40*Δ cells constitutively expressing the mCherry-tagged fusion proteins (X-mCherry) were treated with rapamycin for 8 or 24 h. Expression levels of the fusion proteins and degradation of Sec63-GFP were analyzed by immunoblotting using antibodies against mRFP and GFP, respectively. GFP’, GFP fragments generated by vacuolar cleavage of Sec63-GFP. The AIM mutants (AIM mut) contain the Y242A and M245A mutations. **d** Cells expressing GFP-Atg8 and mCherry-tagged fusion proteins were analyzed by fluorescence microscopy. The images are maximum-intensity projections of *Z* stacks (seven plane stacks, 0.2-μm spacing). X-mCherry puncta that colocalized with GFP-Atg8 were counted, and the results of quantification are shown as means ± s.d. (*n* = 3). ***P = 1.19 × 10^−4^ (TMD-40C), **P = 0.0013 (TMD-GST-40C), ***P = 9.29 × 10^−5^ (TMD-CC^dimer^-40C), **P = 0.0018 (TMD-CC^trimer^-40C) (unpaired two-tailed Student’s *t* test). **e** Cells treated with rapamycin were subjected to FRAP analysis. The images are taken before and after photobleaching of the DsRed-HDEL fluorescence at the indicated region (arrowheads). Fluorescence intensity of DsRed-HDEL at the indicated region was measured, and the results of quantification are shown as means ± s.d. (*n* = 7). The experiments were repeated independently twice (**b**, **c**). Scale bars, 5 μm (**d**), 2 μm (**e**).

Atg40 contains an AIM in the C-terminal cytoplasmic region following the reticulon-like domain (Fig. 1a). The interaction of Atg40 with Atg8 via this AIM is important for ER-phagy^7^. To examine the significance of the Atg40 reticulon-like domain in ER-phagy, we fused the C-terminal region (194–256) of Atg40 to a transmembrane domain (TMD) derived from Sec71, which is sufficient for localization to the ER^19^ (TMD-40C) (Fig. 2a). We induced ER-phagy with rapamycin and evaluated its activity by monitoring GFP fragments (GFP’) generated by vacuolar cleavage of the ER membrane protein Sec63 fused with GFP^7^. Although TMD-40C could not support ER-phagy in *atg40*Δ cells (Fig. 2b), its interaction with Atg8 was weaker than that of Atg40 (Supplementary Fig. 2a). Because Atg40 forms a homodimer (see below), we hypothesized that dimerization of Atg40 increases its affinity for Atg8. Hence, we integrated dimerization modules, glutathione S-transferase (GST) or the coiled-coil region of Gcn4 (CC^dimer^), or a trimer-forming mutant of this region (CC^trimer^) into TMD-40C (TMD-GST-40C, TMD-CC^dimer^-40C, and TMD-CC^trimer^-40C)^20^ (Fig. 2a). These fusion proteins interacted with Atg8 similarly to Atg40 (Supplementary Fig. 2a), but did not rescue ER-phagy in *atg40*Δ cells (Fig. 2b). Thus, targeting the C-terminal region of Atg40 to the ER and its interaction with Atg8 are not sufficient for ER-phagy.

Next, we fused the C-terminal region of Atg40 to the C termini of the reticulon-like proteins Yop1 and Rtn1, yielding Yop1-40C and Rtn1-40C, respectively. Remarkably, both Yop1-40C and Rtn1-40C, but neither Yop1 nor Rtn1, restored ER-phagy in *atg40*Δ cells (Fig. 2c). Introducing mutations (Y242A M245A) into the AIM in Yop1-40C and Rtn1-40C (Yop1-40C^AIM mut^ and Rtn1-40C^AIM mut^, respectively) abolished rescue of ER-phagy by these fusion proteins (Fig. 2c). Thus, the N-terminal region of Atg40, which includes the reticulon-like domain, is replaceable by reticulon-like proteins that are not involved in ER-phagy. Taken together, these results suggest that the reticulon-like function of Atg40 is important for ER-phagy.

### The Atg40 reticulon-like domain is involved in ER fragmentation

mCherry-tagged TMD-GST-40C, TMD-CC^dimer^-40C, and TMD-CC^trimer^-40C were uniformly distributed in the ER under normal conditions, but formed puncta without being transported to the vacuole when cells were treated with rapamycin (Fig. 2d; Supplementary Fig. 2b). These puncta colocalized with the ER lumen marker DsRed-HDEL and puncta of GFP-Atg8 (Fig. 2d, e), suggesting that the fusion proteins were enriched in contact sites between the ER and the isolation membrane. When DsRed-HDEL at these sites was photobleached, fluorescence was immediately recovered, suggesting that these ER regions were not separated from other parts of the ER membrane network (Fig. 2e). Therefore, it is likely that these non-reticulon-like chimeras are capable of tethering the ER to forming autophagosomal membranes through their binding to Atg8, but cannot cause fission of the ER. These results suggest that the reticulon-like domain of Atg40 is important for ER fission to allow sequestration of ER fragments into autophagosomes during ER-phagy.

### Atg8-mediated Atg40 assembly at ER-isolation membrane contacts

**Fig. 3:**
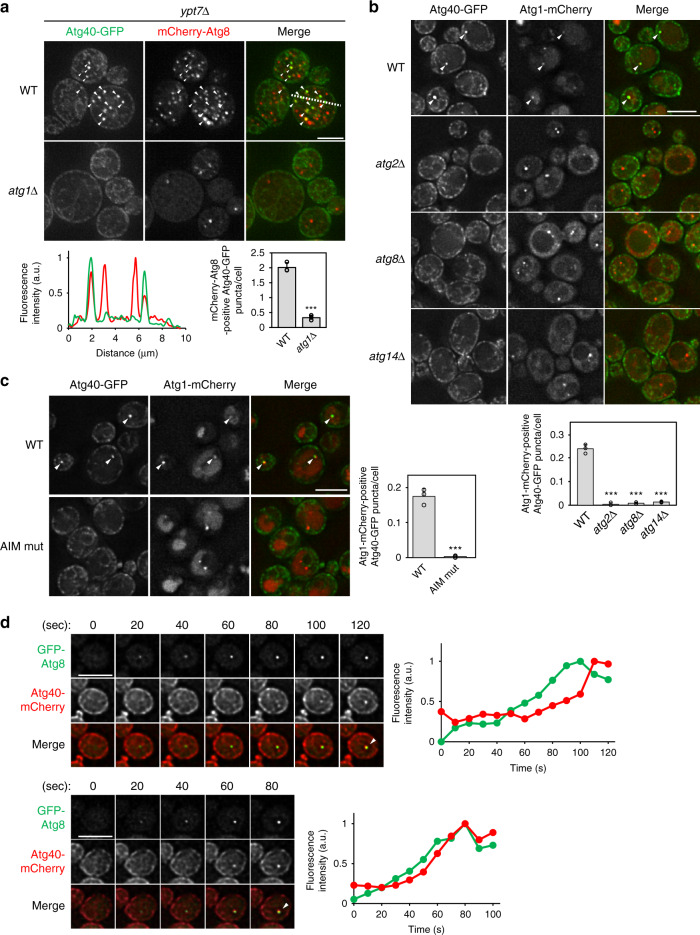
Atg40 is assembled at ER-isolation membrane contact sites through its interaction with Atg8. **a** *ypt7*Δ cells coexpressing Atg40-GFP and mCherry-Atg8 were treated with rapamycin for 6 h, and observed under a fluorescence microscope. The images are maximum-intensity projections of *Z* stacks (seven plane stacks, 0.2-μm spacing). Atg40-GFP puncta that colocalized with mCherry-Atg8 (arrowheads) were counted, and the results of quantification are shown as means ± s.d. (*n* = 3). Line scan analysis of dashed line shows the normalized fluorescence intensity. **b**, **c** Cells were treated with rapamycin for 3 h and observed under a fluorescence microscope. Atg40-GFP puncta that colocalized with Atg1-mCherry (arrowheads) were counted, and the results of quantification are shown as means ± s.d. (*n* = 3). **d** Cells were treated with rapamycin for 15 min and observed under a fluorescence microscope. Images were taken at 10-s intervals. Fluorescence intensity of GFP-Atg8 and Atg40-mCherry puncta are shown (see “Methods”). ***P = 8.02 × 10^−5^ (**a**), ***P = 3.67 × 10^−5^ (b, *atg2*Δ), ***P = 3.44 × 10^−5^ (b, *atg8*Δ), ***P = 3.55 × 10^−5^ (b, *atg14*Δ), ***P = 1.91 × 10^−4 ^ (**c**) (unpaired two-tailed Student’s *t* test). Scale bars, 5 μm.

It was not known whether ER fragments are first generated and then sequestered into the autophagosome, or whether the isolation membrane instead “bites off” a part of the ER network. To investigate the process of ER fragmentation, we analyzed the intracellular dynamics of Atg40 during ER-phagy. Fluorescence microscopy revealed that ER fragments enriched in Atg40 were enclosed within autophagosomes represented by puncta of Atg8 in cells lacking *YPT7* essential for autophagosome–vacuole fusion. However, enrichment of Atg40 in the ER was not observed when autophagosome formation was abolished by deletion of *ATG1* (Fig. 3a). These results are consistent with the idea that multiple Atg40 molecules assemble in the ER membrane in association with autophagosome formation. Atg40 formed puncta in wild-type cells, which colocalized with Atg1 puncta representing the pre-autophagosomal structure (PAS), and these Atg40 puncta disappeared in cells lacking genes essential for autophagosome formation (*ATG2*, *ATG8*, and *ATG14*) (Fig. 3b). In addition, Atg40-mCherry expressed under the control of the constitutive *ADH1* promoter was uniformly distributed in the ER under normal conditions, but assembled and colocalized with GFP-Atg8 upon treatment of the cells with rapamycin (Supplementary Fig. 3a). This suggests that the assembly of Atg40 in the ER requires formation of autophagosomal membranes. Moreover, mutations in the AIM of Atg40 (Y242A M245A) or AIM-binding pocket of Atg8 (P52A R67A) impaired Atg40 puncta formation (Fig. 3c; Supplementary Fig. 3b), although these mutations do not affect autophagosome formation^21^, suggesting that Atg8 mediates the assembly of Atg40 in the ER membrane. Consistent with this, time-lapse microscopy revealed that the fluorescence intensity of Atg40 puncta increased concurrently with that of Atg8 puncta (21 of 22 Atg40 assembly events) (Fig. 3d). These results are consistent with the idea that Atg40 is trapped and assembled through the interaction with Atg8 at ER-isolation membrane contact sites, and subsequently causes ER fragmentation through its reticulon-like function.

### Atg8-mediated assembly of Atg40 drives local ER deformation

**Fig. 4:**
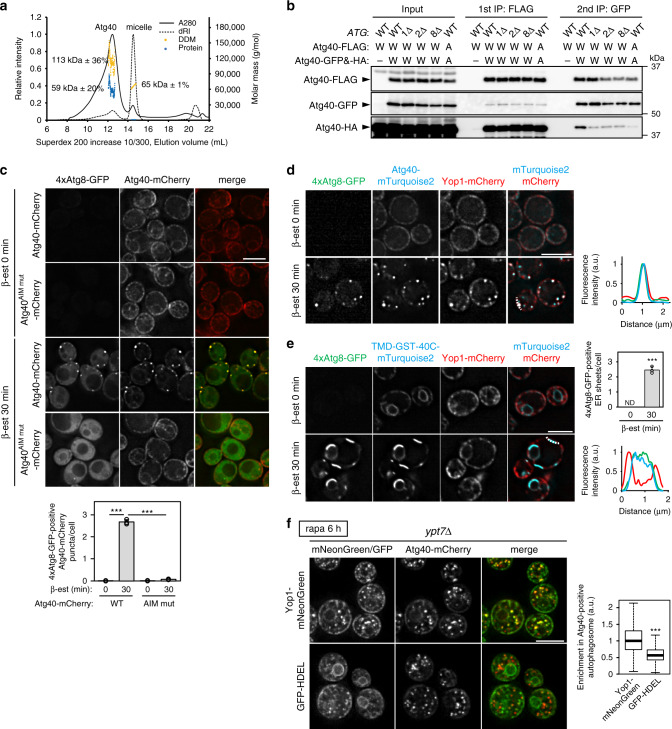
Super-assembly of Atg40 generates ER membrane condensates with highly curved regions. **a** Purified Atg40-6His (30.6 kDa) solubilized in n-dodecyl-β-d-maltoside (DDM) was subjected to SEC-MALS analysis (see “Methods”). dRI differential refractive index. **b** Cells coexpressing Atg40-FLAG, Atg40-GFP, and Atg40-HA under the *ATG40* promoter were treated with rapamycin for 6 h, and cell lysates (input) were subjected to immunoprecipitation using anti-FLAG antibody-conjugated beads. Bound proteins were eluted with the FLAG peptide, and the eluates were subjected to a second immunoprecipitation using anti-GFP antibody. W, wild-type Atg40; A, the AIM mutant of Atg40. The experiments were performed independently three times. **c** Cells constitutively expressing Atg40-mCherry under the *ADH* promoter were treated with β-estradiol to induce expression of GFP-fused, tandemly repeated Atg8^G116A^ (4 × Atg8-GFP), and analyzed by fluorescence microscopy. The quantification results are shown as means ± s.d. (*n* = 3). **d**, **e** Assembly of Atg40-mTurquoise2 (**c**) or TMD-GST-40C-mTurquoise2 (**d**) was induced by expressing 4×Atg8-GFP. Enrichment of Yop1-mCherry in the assemblies was analyzed by fluorescence microscopy. Line scan analysis of dashed line shows the normalized fluorescence intensity. The quantification results are shown as means ± s.d. (*n* = 3). **f** Enrichment of Yop1-mNeonGreen and GFP-HDEL in Atg40-positive autophagosomes was analyzed by fluorescence microscopy. The images are maximum-intensity projections of *Z* stacks (11 plane stacks, 0.2-μm spacing). Box plots (center line, median; box limits, upper and lower quartiles; whiskers, 1.5 × interquartile range) show Yop1-mNeonGreen/GFP-HDEL signals in those autophagosomes against the entire signals (see “Methods”). ***P = 1.68 × 10^−6^ (**c**, WT 0 min vs WT 30 min), ***P = 1.91 × 10^−6^ (**c**, WT 30 min vs AIM mut 30 min), ***P = 4.58 × 10^−5^ (**e**) (unpaired two-tailed Student’s *t* test); ***P = 2.2 × 10^−16^ (**f**) (unpaired two-tailed Mann–Whitney *U* test). Scale bars, 5 μm.

Rtn1 and Yop1 form oligomers to generate membrane curvature^2,22^. Given that Atg40 is assembled at ER-isolation membrane contact sites, we hypothesized that oligomerization of Atg40 locally occurs at these ER subdomains. Size-exclusion chromatography with multiangle light-scattering (SEC-MALS) analysis of purified Atg40 showed that Atg40 intrinsically forms a dimer (Fig. 4a), suggesting that Atg40 dimers assemble into higher-order oligomers at ER-isolation membrane contact sites. To test this possibility, we performed two-step immunoprecipitation using cells simultaneously expressing three differently tagged versions of Atg40 (Atg40-FLAG, Atg40-GFP, and Atg40-HA). In wild-type cells, Atg40-HA, as well as Atg40-FLAG and Atg40-GFP, were present in immunoprecipitates obtained by successive immunoprecipitation with antibodies against FLAG and GFP, suggesting that Atg40 forms oligomers larger than dimers (Fig. 4b). Blocking autophagosome formation by deletion of *ATG1*, *ATG2*, or *ATG8* did not affect Atg40 dimerization (as assessed by coprecipitation of Atg40-GFP and Atg40-HA in the first immunoprecipitation using anti-FLAG antibody), but significantly decreased formation of larger oligomers (Fig. 4b). In addition, oligomerization of Atg40, but not dimerization, was impaired when the AIM was mutated in all the three tagged variants (Fig. 4b). This suggests that most Atg40 exists as a dimer independently of autophagosome formation, but binds Atg8 and forms large assemblies at ER-isolation membrane contact site. Because lipidated Atg8 also forms multimers^23^, it is likely that Atg40 dimers assemble in the ER membrane through multivalent interactions with Atg8 multimers in the isolation membrane.

Next, we investigated the effects of Atg8-mediated super-assembly of Atg40 on ER membrane dynamics. To artificially induce Atg40 assembly, a quadruple repeat of Atg8 C-terminally fused with GFP (4 × Atg8-GFP), in which the G116A mutation was introduced into the Atg8 sequences to prevent Atg4-mediated cleavage, was expressed under the control of the β-estradiol-inducible system^24^ in cells constitutively expressing Atg40-mCherry. Atg40-mCherry formed punctate structures with 4 × Atg8-GFP immediately after its induction (addition of β-estradiol) (Fig. 4c). By contrast, the AIM mutant of Atg40-mCherry did not form these structures, and 4 × Atg8-GFP was dispersed throughout the cytoplasm, demonstrating that the interaction of Atg40 with 4 × Atg8-GFP led to the formation of these structures. Clustering of Atg40 was also induced by expression of Atg8 fused with a coiled-coil sequence that forms a tetramer (Atg8-CC^tetramer^) (Supplementary Fig. 4a)^25,26^. Remarkably, Yop1 and Rtn1, but not DsRed-HDEL, were enriched in the 4 × Atg8- and Atg40-positive structures (Fig. 4d; Supplementary Fig. 4b), suggesting that highly curved ER membranes are tightly packed in these structures. We also showed that TMD-GST-40C, which efficiently interacts with Atg8 but does not have a reticulon-like domain (Fig. 2), was also assembled upon induction of 4 × Atg8-GFP expression. However, unlike Atg40, this protein formed large sheets with 4 × Atg8-GFP in the ER membrane (Fig. 4e; Supplementary Fig. 4c). Yop1 and Rtn1 were not enriched in these sheet regions, suggesting that the assembly of TMD-GST-40C failed to fold the ER. These results suggest that Atg8-mediated super-assembly of Atg40 enhances its reticulon-like function, leading to the folding of the ER, which in turn promotes its efficient loading into the autophagosome. Consistent with this idea, previous electron microscopy revealed that compactly folded ER membranes are enclosed within autophagosomes^7^. We also showed that Yop1 is highly enriched in Atg40-positive autophagosomes compared with GFP-HDEL (Fig. 4f).

Our results suggested that super-assemblies of Atg40 form through interactions between Atg40 dimers and Atg8 multimers. Multivalent protein–protein interaction drives liquid–liquid phase separation^27^. When super-assembly of Atg40 was induced by 4 × Atg8-GFP, the fluorescence of 4 × Atg8-GFP within Atg40 assemblies recovered swiftly after photobleaching, suggesting that 4 × Atg8-GFP within the assemblies are exchangeable with molecules outside the assemblies (Supplementary Fig. 4d). We further tested whether Atg8-CC^tetramer^ mediates super-assembly of GST-40C (dimer) using recombinant proteins in vitro. Mixing mCherry-Atg8-CC^tetramer^ with SNAP-tagged GST-40C, which was labeled with the fluorescent dye Alexa Fluor 488 in the SNAP tag, induced formation of spherical condensates (Supplementary Fig. 5a). In contrast, condensate formation was not observed when SNAP-GST-40C^AIM mut^ or mCherry-Atg8 without a tetramerization sequence was used (Supplementary Fig. 5a). The droplets slowly coalesced with each other (Supplementary Movie 1), and fluorescence recovery after photobleaching (FRAP) analysis showed ~36% fluorescence recovery of SNAP-GST-40C within 200 s (Supplementary Fig. 5b). These data suggested that tetramerized Atg8 induced phase separation of GST-40C to form semi-liquid droplets in vitro, which is mediated by multivalent interactions between these proteins. Taken together, these results raise the possibility that super-assembly of Atg40 is driven by two-dimensional liquid–liquid phase separation at the interface between the ER and isolation membrane.

### Structural basis of ER-phagy receptor binding to Atg8s

**Fig. 5:**
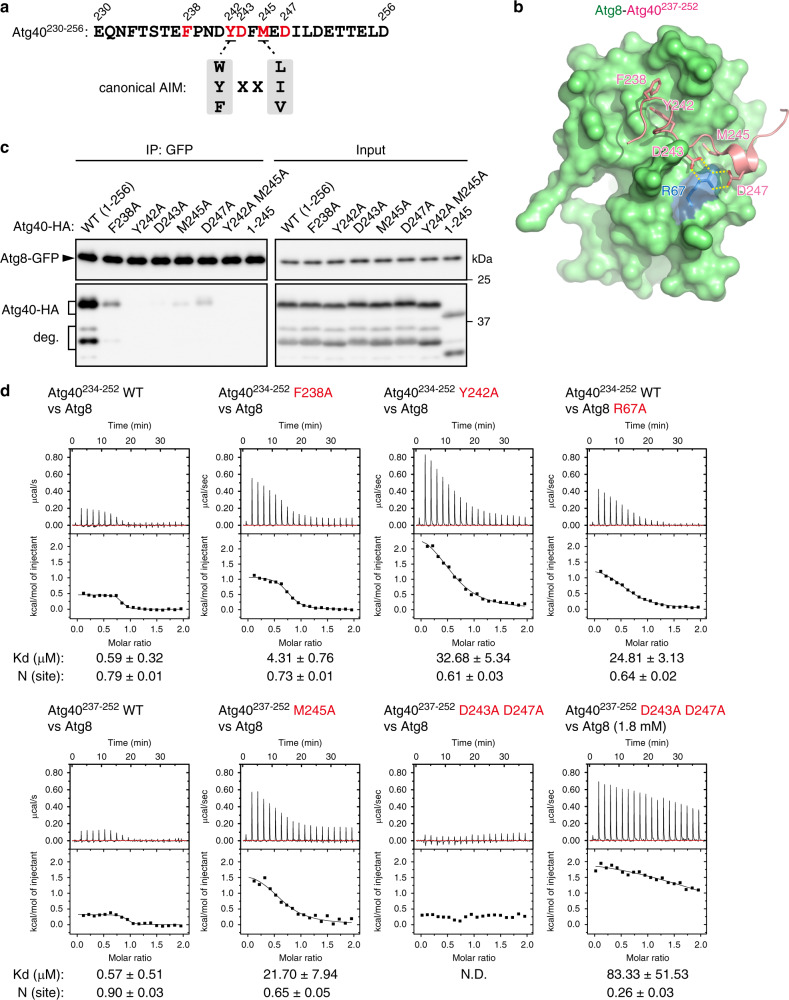
The structural basis of the Atg40–Atg8 interaction. **a** Amino-acid sequences of the canonical AIM and the C-terminal region of Atg40 (230–256). The residues colored red were replaced with alanine in **c**. **b** Crystal structure of Atg8-Atg40^237–252^. Yellow dashed lines, salt bridges. **c** *ATG8-GFP atg4*Δ *atg40*Δ cells expressing Atg40-HA were treated with rapamycin for 6 h, and the cell lysates (input) were subjected to immunoprecipitation using anti-GFP antibody. The immunoprecipitates (IP) are analyzed by immunoblotting using antibodies against GFP and HA. Additional bands in Atg40-HA immunoblotting represent its degradation products (deg.). The experiments were repeated independently twice. **d** Binding affinity of Atg40 peptides to Atg8 was measured by ITC.

The core of the AIM is composed of four amino-acid residues [W/F/Y]-X-X-[L/I/V]^28^ (Fig. 5a). The first aromatic residue and the fourth aliphatic residue bind to two hydrophobic pockets in Atg8 (the W- and L-sites, respectively), and the main chain of the motif forms an intermolecular parallel β-sheet with a β-strand (β2) of Atg8^28^. To elucidate the structural basis of the Atg40–Atg8 interaction, we determined the crystal structure of Atg8 N-terminally fused with the C-terminal sequence of Atg40 (Atg40^237–252^) containing the AIM (Y^242^-D-F-M) (Fig. 5a, b; Supplementary Fig. 6a). As with canonical AIMs, the first and fourth residues Y242 and M245 bound to the W- and L-sites of Atg8, respectively. In addition, the second residue D243 formed salt bridges with the R67 residue of Atg8 (Fig. 5b; Supplementary Fig. 6b). Moreover, the Atg40 region C-terminal to M245 assumed a helical conformation, and D247 in this helix formed salt bridges with R67 of Atg8. Residue F238 on the N-terminal side of the core motif also interacted with I21 and the aliphatic portion of R20 of Atg8. Co-immunoprecipitation analysis revealed that, like the core residues^7^, the residues that interacted with Atg8 in the crystal structure were crucial for Atg40 binding to Atg8 in yeast cells (Fig. 5c). We also examined the interactions of Atg40 peptides with Atg8 by isothermal titration calorimetry (ITC). We prepared two “wild-type” peptides, Atg40^234–252^ and Atg40^237–252^, because the former peptide with the M245A and D243A D247A mutations could not be synthesized. Both Atg40^234–252^ and Atg40^237–252^ bound Atg8 with similar Kd values (0.59 ± 0.32 μM and 0.57 ± 0.51 μM, respectively). These interactions were markedly impaired by the D243A, D247A, and F238A mutations, as well as the Y242A and M245A mutations in the Atg40 peptides or the R67A mutation in Atg8 (Fig. 5d). Thus, these additional interactions increase Atg40 affinity to Atg8. Importantly, ER-phagy was defective in cells expressing the D243A, D247A, and F238A mutants of Atg40 (Supplementary Fig. 6c), demonstrating that enhancement of Atg40 binding to Atg8 by these additional interactions is required for ER-phagy. When the C-terminal region of Atg40 containing the AIM (residues 240–256) was swapped with the AIM of the autophagy receptors Atg19 and Atg34^29,30^ (Supplementary Fig. 6d), the interaction with Atg8 was significantly decreased, resulting in impairment of ER-phagy (Supplementary Fig. 6e, f). These results suggest that strong binding to Atg8 is important for execution of ER-phagy by Atg40.

**Fig. 6:**
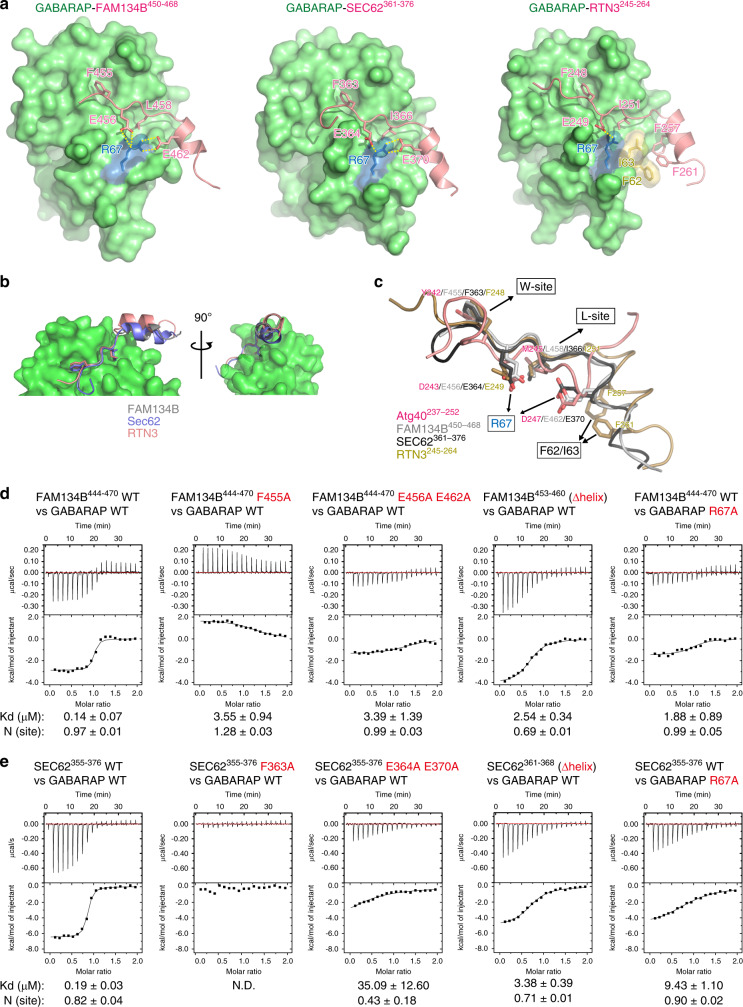
Evolutionary conserved helices C-terminal to the AIM/LIR enhance interactions between ER-phagy receptors and Atg8-family proteins. **a**, **b** Crystal structures of GABARAP-FAM134B^450–468^, GABARAP-SEC62^361–376^, and RTN3^245–264^. Green and pink indicate GABARAP and ER-phagy receptor AIMs, respectively. **c** Superimposition of Atg8/GABARAP-binding regions of ER-phagy receptors based on the structures of Atg8 and GABARAP. **d**, **e** Binding affinity of FAM134B (**d**) and SEC62 (**e**) peptides to GABARAP was measured by ITC.

We also determined the crystal structures of GABARAP (a mammalian Atg8 homologue) bound to AIM (LIR)-containing sequences from the mammalian ER-phagy receptors FAM134B, SEC62, and RTN3 (FAM134B^450–468^, SEC62^361–376^, and RTN3^245–264^) (Fig. 6a, b). For the analysis of RTN3, which possesses six AIMs, we used the third AIM from the N terminus, because it is well conserved and especially important for RTN3 binding to mammalian Atg8-family proteins (mAtg8s)^9^. Remarkably, all the AIM-containing regions of the three ER-phagy receptors are structurally similar to that of Atg40, consisting of the core AIM and its C-terminal short helix. As reported previously^8^, the first and fourth AIM residues of FAM134B (F455 and L458) bound to the W- and L-sites in GABARAP (Fig. 6a, c; Supplementary Fig. 7). (Of note, an FAM134B sequence used in the previous structural analysis^8^ did not contain the C-terminal short helical region.) The core AIM residues of SEC62 (F363 and I366) and RTN3 (F248 and I251) interacted with GABARAP in a similar manner (Fig. 6a, c; Supplementary Fig. 7). In addition, as with Atg40, the second AIM residues (E456 of FAM134B, E364 of SEC62, and E249 of RTN3) and acidic residues in the short helices (E462 of FAM134B and E370 of SEC62) interacted with R67 of GABARAP (Fig. 6a, c; Supplementary Fig. 7). The short helix of RTN3^245–264^ lacks an acidic residue and instead possesses hydrophobic residues (F257 and F261), which formed hydrophobic interactions with Phe62 and Ile63 of GABARAP. In ITC analyses, FAM134B^444–470^ and SEC62^355–376^ bound to GABARAP-subfamily proteins (GABARAP, GAPARAP-L1, and GABARAPL2) with Kd ranging from 0.11 to 0.30 μM and from 0.19 to 0.57 μM, and to LC3-subfamily proteins (another subfamily of mAtg8s, LC3A, LC3B, and LC3C) with Kd ranging from 0.21 to 1.11 μM and from 0.92 to 5.18 μM, respectively (Fig. 6d, e; Supplementary Figs. 8a, c, 9a, b), suggesting that these AIMs interact with mAtg8s with low specificity. These interactions were significantly weakened by mutations at E456 and E462 in FAM134B^444–470^ and E364 and E370 in SEC62^355–376^, as well as at the core AIM residues in these peptides, or deletion of the C-terminal helices (Δhelix) (Fig. 6d, e; Supplementary Figs. 8 and 9). In addition, the interactions were also impaired by alanine substitution for R67 in GABARAP and R70 in LC3B (Fig. 6d, e; Supplementary Fig. 9a, b). Wild-type RTN3^245–264^ also interacted with mAtg8s (Supplementary Fig. 10a). However, the Kd values were substantially larger than those for FAM134B and SEC62 peptides, and the interaction with LC3C was not detected. Strong RTN3 binding to mAtg8s would require a cumulative interaction by its multiple AIMs. Nonetheless, deletion of the short helix from RTN3^245–264^ reduced the interactions with mAtg8s (Supplementary Fig. 10b). Thus, the C-terminal helix-assisted AIM/LIR is a unique structural basis commonly seen in the interactions of yeast Atg40 and these mammalian ER-phagy receptors with Atg8-family proteins.

## Discussion

**Fig. 7:**
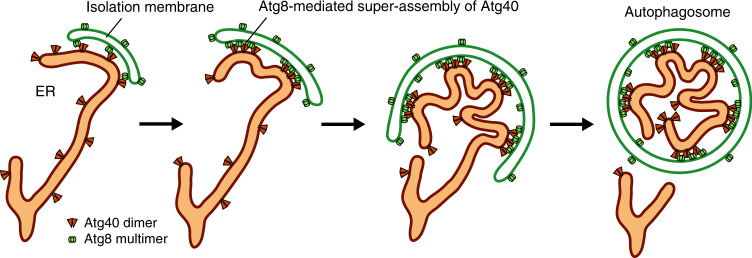
Model for local ER remodeling by Atg40 during ER-phagy. When autophagosome formation initiates in the vicinity of the ER, Atg40 dimers are trapped and assembled at ER-isolation membrane contact sites through the interaction with Atg8 multimers on the isolation membrane. The resulting super-assembly of Atg40 bends the ER membrane with the reticulon-like function, leading to efficient ER packing into the autophagosome.

The mechanism of the local remodeling of the ER in its autophagic sequestration is an important question in the study of ER-phagy. Here, we showed that the ER-phagy receptor Atg40 forms a super-assembly in association with Atg8 at contact sites between the ER and isolation membrane, resulting in local ER remodeling in which the reticulon-like domain of Atg40 bends ER membranes to promote efficient packing of ER fragments into autophagosomes (Fig. 7). Unlike other reticulon-like proteins such as Yop1 and Rtn1, which constitutively act to shape overall ER structures, Atg40 locally exerts its membrane-remodeling function in ER regions fated for sequestration into autophagosomes. Super-assembly of Atg40, induced by its interaction with Atg8 at ER-isolation membrane contact sites, is an elaborate mechanism that enables the cell to meet this functional demand.

Recent work suggested that FAM134B generates membrane curvature and senses curvature with its reticulon-like domain, leading to its enrichment in curved regions^31^. Because Atg40 prefers highly curved ER regions^7^, the same principle could serve to increase local concentrations of Atg40, promoting super-assembly.

Yop1 and Rtn1 fused with the AIM-containing C-terminal region of Atg40 restored ER-phagy in *atg40*Δ cells (Fig. 2c). By contrast, non-reticulon-like ER membrane proteins fused with the same region of Atg40 accumulated at autophagosome formation sites, but did not cause fission of the ER (Fig. 2d, e). In mammalian cells, RTN3L engineered to oligomerize caused ER fragmentation^9^. Moreover, the recombinant protein of FAM134B decreased the size of liposomes, in which its reticulon-like domain plays a crucial role^8,31^. These results suggest that the reticulon-like domain of these ER-phagy receptors is involved in not only ER folding but also ER fission for sequestration into the autophagosome. However, it remains to be clarified whether the reticulon-like domain of Atg40 directly drives ER fission, or facilitates an event caused by other factors. Nonetheless, our data strongly suggest that ER fragmentation occurs in concert with autophagosome formation.

The fourth residue of the Atg40 AIM is methionine (M245), but peptide array experiments using several AIM/LIR-containing sequences showed that methionine is not tolerated at the fourth position of the AIM/LIR^32–34^. Our structural analysis revealed that residue M245 bound properly to the L-site on Atg8; thus Atg40 is the first example of a protein in its class that uses methionine at the fourth position of the AIM. In the canonical AIM exemplified by Atg19^29^, the main chain in the AIM core region forms an intermolecular β-sheet with β2 of Atg8 (Supplementary Fig. 11). However, in Atg40, the main chain at M245 was included in the C-terminal helix, and thus distant from Atg8, enabling accommodation of methionine’s long side chain in the L-site. A similar structural basis was seen in a recently discovered, non-canonical AIM named the helical AIM^35^. Thus, our results provide important insights into the diversity of the AIM/LIR core sequence.

We found that acidic residues at the second position of the AIM and a short helix C-terminal to the AIM substantially strengthen the Atg40–Atg8 interaction. The mammalian ER-phagy receptors FAM134B, SEC62, and RTN3 interacted with Atg8-family proteins in a similar manner. As with these ER-phagy receptors, FYCO1, which links autophagosomes to kinesin motors, and super-strong Atg8-biding peptides derived from giant ankyrins also use acidic residues in α-helices C-terminal to the AIM/LIR to bind Atg8-family proteins^33,36,37^. Thus, this mode of interaction is likely to be common among proteins that require tight Atg8 binding. Indeed, our mutational analysis revealed that Atg8 binding, enhanced by additional interactions, is important for execution of ER-phagy by Atg40. Strong Atg8 binding is likely to be required for not only tight linking of the ER to expanding isolation membranes but also for Atg8-mediated super-assembly of Atg40, which leads to local remodeling of the ER.

In conclusion, our findings reveal that folding and fission of the ER occurs in conjunction with autophagosome biogenesis during ER-phagy, and that this process involves Atg8-mediated super-assembly of the reticulon-type ER-phagy receptor Atg40 at ER-isolation membrane contacts. In selective autophagy of mitochondria and the nucleus, these organelles also undergo fission to generate small fragments, but the mechanisms underlying this organelle fragmentation still remain elusive^7,38^. This study also provides important insights into elucidating these mechanisms.

## Methods

### Yeast strains

Yeast strains used in this study are derivatives of BY4741, W303-1A, or BJ3505^39–41^, and are listed in Supplementary Table 1. Gene disruption and gene tagging were performed by a standard PCR-based method^42^. pRS303- and pRS306-based plasmids and YIPlac204TKC-DsRed-Express2-HDEL were linearized with the appropriate restriction enzymes and integrated into the *HIS3*, *URA3*, or *TRP1* locus, respectively^42,43^.

### Media

Yeast cells were cultured at 30 °C in YPD medium (1% yeast extract, 2% peptone, and 2% glucose) or SD + CA medium (0.17% yeast nitrogen base without amino acids and ammonium sulfate), 0.5% ammonium sulfate, 0.5% casamino acids, and 2% glucose supplemented with 0.002% adenine, 0.002% uracil, and 0.02% or 0.008% tryptophan appropriately. To induce ER-phagy, cells grown to mid-log phase were treated with 200 ng ml^−1^ rapamycin. For 4 × Atg8-GFP expression, cells grown to mid-log phase were treated with 1 μM β-estradiol. Cells expressing Atg40-mCherry or 40C-tagged proteins under the control of the *CUP1* promoter were cultured in SD + CA medium containing 250 μM CuSO_4_ overnight, and then treated with rapamycin or β-estradiol. In Fig. 2d and Supplementary Fig. 2b, cells were grown in SD + CA medium containing 50 μM CuSO_4_. For growth assays, cells grown to mid-log phase were spotted onto YPD agar plates containing 0.5 mM IAA or not, and incubated at 30 °C for 1 day.

### Plasmids

Plasmids used in this study are listed in Supplementary Table 2. These plasmids were generated by assembling appropriate DNA fragments using the Gibson Assembly method (New England Biolabs). Point mutations were introduced using the QuikChange site-directed mutagenesis kit (Agilent), and oligonucleotides listed in Supplementary Table 3. YIPlac204TKC-DsRed-Express2-HDEL was purchased from Addgene (Plasmid #21770). A yeast strain expressing the β-estradiol-inducible transcription factor Z4EV and a plasmid encoding the Z4 promoter were gifts from Dr. Yasuhiro Araki.

### Fluorescence microscopy

Three different microscopy systems were used in this study, as described below. All images were processed and analyzed using the Fiji software^44^.

System 1: A Delta Vision Elite microscope system (GE Healthcare) equipped with a scientific CMOS camera (pco.edge 5.5; PCO AG) and a ×60 objective lens (PLAPON, NA/1.42; Olympus). Images were acquired and deconvoluted using SoftWoRx software. The images in Figs. 2d, 3a-c, and 4c-f and Supplementary Figs. 2b, 3, and 4a-c were acquired using this system.

System 2: An inverted microscope (IX81; Olympus) equipped with an electron-multiplying CCD camera (ImagEM C9100-13; Hamamatsu Photonics), a ×150 objective lens (UAPON 150XOTIRF, NA/1.45; Olympus), a Z drift compensator (IX3-ZDC2; Olympus), 488-nm and 588-nm lasers (Coherent), a dichroic mirror reflecting 405-nm, 488-nm, and 588-nm wavelength (Olympus). GFP and mCherry fluorescence was separated by the DV2 multichannel imaging system (Photometrics) equipped with a Di02-R594-25 × 36 dichroic mirror (Semrock), a TRF59001-EM ET band-pass filter (Chroma), and a FF01-624/40-25 band-pass filter (Semrock). The images in Figs. 1b and 3d and Supplementary Fig. 1b, c were acquired using this system and MetaMorph software (Molecular Devices). In time-lapse imaging, cells in the glass-bottom dish were kept at 30 °C using a stage top incubator (TOKAI HIT). The images in Fig. 3d were acquired by deconvolution using AutoQuant X3 software (Media Cybernetics).

System 3: A confocal microscope system (LSM780; Carl Zeiss) equipped with a ×63 objective lens (Plan-Apochromat, NA/1.40; Carl Zeiss). FRAP imaging was performed using this system and the ZEN 2012 software (Carl Zeiss).

### Quantification of fluorescence microscopy images

To count Atg40-GFP puncta that colocalized with Atg1-mCherry puncta (Fig. 3b, c), Supplementary ImageJ Script 1 was used. The criterion for colocalization was the presence of maxima of Atg40-GFP puncta within 266 nm (equivalent to two pixels) from those of Atg1-mCherry puncta. Colocalization of GFP puncta and mCherry puncta in Figs. 2d, 3a, and 4c and Supplementary Fig. 3 was counted using Supplementary ImageJ Script 1, in which noise score was adjusted depending on the brightness of an image of each experiment. In Fig. 3d, fluorescence intensity of GFP-Atg8 and Atg40-mCherry was shown as a maximum value of respective fluorescent intensities within 326 nm from the center of GFP-Atg8 puncta. The number of 4 × Atg8-GFP-positive ER sheets in Fig. 4e was manually counted. Fluorescence intensities along dashed lines were measured using Fiji. To measure ER protein enrichment in autophagosomes (Fig. 4f), fluorescence images of cells expressing Atg40-mCherry and Yop1-mNeonGreen or GFP-HDEL were analyzed using the Fiji software as follows. The fluorescence intensities of Yop1-mNeonGreen and GFP-HDEL colocalized with Atg40-mCherry-positive autophagosomes, as well as the total fluorescence intensities of Yop1-mNeonGreen and GFP-HDEL were measured using Supplementary ImageJ Scripts 2 and 3, respectively, from which the background values were subtracted. The former values were divided by the latter values in the individual images, and data are presented relative to the median of the Yop1-mNeonGreen intensity (defined as 1).

### Immunoblotting

Frozen yeast cells were treated with 20% trichloroacetic acid on ice for 15 min, spun down, washed with ice-cold acetone, resuspended in urea SDS sample buffer (100 mM MOPS-KOH (pH 6.8), 4% SDS, 100 mM DTT, and 8 M urea), and incubated at 65 °C for 10 min. The samples were homogenized using 0.5-mm YZB zirconia beads (Yasui Kikai) and a FastPrep-24 (MP Biomedicals). These samples were subjected to SDS-PAGE, followed by immunoblotting using antibodies against GFP (Clontech, 632381), HA (Roche, 11867431001), FLAG (Sigma, F1804), mRFP, and Kar2 (gifts from Dr. Toshiya Endo).

### Immunoprecipitation

For immunoprecipitation of Atg8-GFP, yeast cells were disrupted in IP buffer (50 mM Tris-HCl (pH 8.0), 150 mM NaCl, 1 mM EDTA, and 10% glycerol) containing 2 mM phenylmethylsulfonyl fluoride (PMSF) and 2× complete protease inhibitor cocktail (PIC) (Roche) using a multi-beads Shocker (Yasui Kikai) and 0.5-mm YZB zirconia beads. The lysates were solubilized with 0.5% Triton X-100 and subjected to centrifugation at 15,000 *g* for 15 min. The supernatant was incubated with GFP nanobody-conjugated magnetic beads^45^ for 2 h at 4 °C. After the beads were washed three times with IP buffer containing 0.3% Triton X-100, the bound proteins were eluted with urea SDS sample buffer.

In two-step immunoprecipitation of Atg40, yeast cells were spheroplasted by incubation in 0.5 × YPD containing 1 M sorbitol and 200 μg ml^−1^ Zymolyase 100 T (Seikagaku Biobusiness) for 45 min at 30 °C. The spheroplasts were washed with 20 mM HEPES-KOH (pH 7.2) containing 1.2 M sorbitol and incubated in 0.5 × YPD containing 1 M sorbitol and 200 ng ml^−1^ rapamycin for 6 h. After washing with 50 mM Tris-HCl (pH 8.0) containing 150 mM NaCl, 5 mM EDTA, 5 mM EGTA, 50 mM NaF, and 1.2 M sorbitol, the spheroplasts were frozen in liquid nitrogen and stored at −80 °C. The frozen cell pellets were solubilized in IP buffer containing 2 mM PMSF, 2 × PIC, and 0.5% Triton X-100 for 30 min at 4 °C. The lysates were centrifuged at 100,000 *g* for 30 min, and the supernatants were incubated with anti-FLAG antibody-conjugated magnetic beads^46^ for 2 h. After the beads were washed with IP buffer containing 0.3% Triton X-100, the bound proteins were competitively eluted with IP buffer containing 2 mg ml^−1^ 3 × FLAG peptide and 0.3% Triton X-100. The eluates were incubated with the GFP nanobody-conjugated beads for 1.5 h, and the bound proteins were eluted with urea SDS sample buffer.

### Cysteine-modification assay

The spheroplasts were suspended in buffer A (20 mM HEPES-KOH (pH 7.2), 1.2 M sorbitol, 1 mM EDTA and 1 × PIC) and passed through polycarbonate filters with a pore diameter of 3 μm (Merck Millipore) to disrupt cells. The resultant lysates were treated with 5 mM DTT for 15 min at 4 °C and centrifuged at 17,400 *g* for 15 min. The pellets were washed with buffer A, resuspended in buffer A with or without 50 μM methoxy polyethylene glycol maleimide (MW 5000) (JenKem Technology) and 1% Triton X-100, and incubated for 30 min at 30 °C. After the reactions were quenched by addition of 100 mM DTT, samples were analyzed by immunoblotting.

### Plasmid constructions for crystallization and in vitro binding assay

All plasmids for expression of recombinant proteins and peptides in bacteria were based on a pGEX6P-1 vector (GE Healthcare). Construction of pGEX6P-ScAtg8 (1-116) K26P and pGEX6P-rat LC3B (1-120) was previously described^35,47^. All mutations were generated by PCR-based mutagenesis. The genes were amplified by PCR and inserted downstream of the HRV 3C protease recognition site by NEBuilder HiFi DNA Assembly Master Mix (New England Biolabs) except for that encoding human GABARAP (1-116), which was inserted using restriction enzymes for the *Nde*I and *Bam*HI sites. Plasmids for expression of fusion proteins for crystallization were constructed by inserting the genes encoding Atg40^237–252^, human FAM134B^450–468^, human SEC62^361–376^ with the T367D mutation, and human RTN3^245–264^ isoform e into upstream of the sequence encoding Atg8 K26P of pGEX6P-Atg8 (the K26P mutation was introduced for stabilizing Atg8) or upstream of the sequence encoding GABARAP of pGEX6P-GABARAP (with the F3S V4T mutations for enhancing crystallization). For phase-separation experiments, the mCherry and BNDL1^25,26^ sequences were inserted into upstream and downstream of the *ATG8* gene in pGEX6P-Atg8 K26P, respectively. pGEX-SNAP-40C was constructed by the removal of the HRV 3C protease recognition site and the insertion of the SNAP sequence from pSNAP-tag(T7) vector (New England Biolabs) with a linker (Gly–Gly–Gly–Ser–Gly–Gly–Gly) and Atg40^194–256^ following a linker (Ser–Ala–Ser–Ser) to upstream and downstream of GST in pGEX6P-1 vector, respectively. In the construction of plasmids for peptide production, a Tyr residue was attached to their C termini for enabling the quantification by absorbance at 280 nm (Supplementary Table 4).

### Purification of proteins and peptides

Some of peptides (Supplementary Table 4) and all proteins except Atg40-6His were expressed in *E. coli* BL21 (DE3). Bacteria were cultured at 37 °C until OD_600_ became 0.8–1.0 and further cultured at 16 °C with 100 μM IPTG overnight. After centrifugation, the pellets were resuspended with 20 ml PBS with 0.5 mM EDTA and lysed by sonication for 10 min. After centrifugation, the supernatants were incubated with GST-accept resin (Nacalai tesque). After three times wash by PBS, the proteins were eluted with glutathione buffer (10 mM glutathione and 50 mM Tris pH 8.0). The buffer of the eluates was exchanged with PBS by a Bio-Scale Mini BioGel P-6 desalting column (Bio-Rad). After incubation with GST-fused HRV 3 C protease at 4 °C overnight, the samples were subjected to a GST-accept resin column to remove excised GST and the protease. RTN3^245–264^-GABARAP was purified by cation-exchange chromatography using CM Sepharose FF column (GE Healthcare) with 500 mM MES pH 6.0 as buffer B and 50 mM MES pH 6.0 and 1 M NaCl as buffer C. The samples were further subjected to size-exclusion chromatography with 20 mM HEPES pH 6.8, 150 mM sodium chloride except for mCherry-Atg8-CC^tetramer^ (20 mM HEPES pH 6.8, 500 mM sodium chloride) using a Superdex 75 10/300 or Superdex 200 26/60 column. As described in Supplementary Table 4, the Atg40^237–252^ M245A and Atg40^237–252^ D243A D247A peptides were synthesized by Cosmo Bio, and the FAM134B^453–460^ and SEC62^361–368^ peptides were synthesized by BEX Co., Ltd. The Atg40^234–252^ peptide and its variants were prepared using Liberty Blue peptide synthesizer (CEM Corporation). After dissolving these synthesized peptides in 300 μl water with ~10 μl ammonium hydroxide, they were subjected to size-exclusion chromatography with 20 mM HEPES pH 6.8 containing 150 mM sodium chloride using a Superdex 75 10/300 column.

For Atg40-6His purification, the BJ3505 yeast strain expressing Atg40-6His under the control of the *GPD* promoter was grown in YPD until OD_600_ became 4. Harvested cells were resuspended in buffer D (20 mM Tris-HCl (pH 8.0), 150 mM NaCl, 20 mM KCl, 10 mM MgCl_2_, 2 mM PMSF, and 5 mM β-mercaptoethanol) and disrupted using zirconia beads and a Shake Master NEO (Biomedical Science). After removing undisrupted cells by centrifugation, the supernatants were centrifuged at 45,000 rpm for 30 min using a Type 45Ti rotor to spin down membrane fractions. The pellets were washed with buffer E (50 mM Tris-HCl (pH 8.0), 1.15 M NaCl, 20 mM KCl, 10 mM MgCl_2_, 2 mM PMSF, and 5 mM β-mercaptoethanol), resuspended in buffer F (20 mM Tris-HCl (pH 7.5), 0.3 M sucrose, and 0.1 mM CaCl_2_), and stored at −80 °C. After thawing, DDM (1% final), imidazole (20 mM final), PMSF (1 mM final), and protease inhibitor cocktail (Nacalai Tesque, 1 × final) were added to the lysates to solubilize membrane proteins. The lysates were centrifuged at 50,000 g for 45 min and the resultant supernatants were subjected to affinity purification using Ni-NTA agarose (Qiagen). After washing Ni-NTA agarose successively with buffer G (20 mM Tris-HCl (pH 8.0), 1 M NaCl, and 0.03% DDM) and buffer H (20 mM Tris-HCl (pH 8.0), 150 mM NaCl, 75 mM imidazole, and 0.03% DDM), the bound proteins were eluted with buffer I (20 mM Tris-HCl (pH 8.0), 150 mM NaCl, 350 mM imidazole, and 0.03% DDM). After size-exclusion chromatography with buffer J (20 mM Tris-HCl (pH 8.0), 150 mM NaCl, and 0.03% DDM) and concentration, the samples were subjected to SEC-MALS analysis.

### Crystallization

All crystallization trials were performed by the sitting-drop vapor-diffusion method at 20 °C. For crystallization of Atg40^237–252^-Atg8 fusion, 41 mg/ml protein was incubated with 10% PEG 8000, 0.1 M HEPES pH 7.5, 8% ethylene glycol as a reservoir and C6 well of silver bullets bio (0.04% cortisone, 0.04% (±)-epinephrine, 0.04% protoporphyrin disodium salt, 0.04% pyridoxine, 0.04% thymidine 5’-monophosphate disodium salt hydrate, and 0.02 M HEPES buffer pH 6.8) (Hampton Research) as an additive. The volume ratio of protein, reservoir, and additive was 2:1:1. For crystallization of FAM134B^450–468^-GABARAP fusion, 126 mg/ml protein was mixed with a reservoir solution consisting of 1.91 M ammonium sulfate, 0.1 M sodium citrate pH 5.5, and 0.18 M potassium sodium tartrate. For crystallization of SEC62^361–376^-GABARAP fusion, 40 mg/ml protein was mixed with a reservoir solution consisting of 1.5 M ammonium sulfate and 0.1 M Tris pH 8.5. For crystallization of RTN3^245–264^-GABARAP fusion, 61 mg/ml protein was mixed with a reservoir solution consisting of 0.8 M potassium phosphate monobasic, 0.8 M sodium phosphate monobasic, and 0.1 M HEPES pH 7.5. The mixing volume ratio of protein and reservoir solution was 1:1 and mixed solution was equilibrated against the reservoir solution. All crystals were obtained within 4 days.

### Diffraction data collection

Crystals were soaked in cryoprotectant and frozen in liquid nitrogen. The cryoprotectant for Atg40^237–252^-Atg8 fusion was prepared by adding 25% ethylene glycol to the reservoir solution. The cryoprotectants for FAM134B^450–468^-GABARAP and SEC62^361–376^-GABARAP fusions were prepared by adding 33% glycerol to each reservoir solution. The cryoprotectants for RTN3^245–264^-GABARAP fusion were prepared by adding 30% glycerol to 0.6 M potassium phosphate monobasic, 0.6 M sodium phosphate monobasic, and 0.1 M HEPES pH 7.5. The flash-cooled crystals were kept in a stream of nitrogen gas at −178 °C during data collection. Diffraction data collections of the crystals were done by using EIGER X4M detector for Atg40 at the beamline BL-1A, KEK, Japan, Pilatus 2M-F detector for Sec62 at the beamline NE-3A, KEK, Japan, and EIGER X 9 M detector for FAM134B and RTN3 at the beamline BL32XU, SPring-8, Japan, with the wavelength of 1.1000, 1.0000, 1.0000, and 1.0000 Å, respectively. The diffraction data were indexed, integrated, and scaled using XDS^48^. Parameters of diffraction data collection are summarized in Supplementary Table 5.

### Structure determination

The structures of Atg40^237–252^-Atg8, FAM134B^450–468^-GABARAP, SEC62^361–376^-GABARAP, and RTN3^245–264^-GABARAP fusion proteins were determined by the molecular replacement method using Phenix^49^. Atg8 (PDBID: 2ZPN) and GABARAP (PDBID: 1GNU) structures were used as a search model. Crystallographic refinement was done by using Phenix and COOT programs^50^. All structural images in the manuscript were prepared by PyMOL (The PyMOL Molecular Graphics System, Version 2.0 Schrödinger, LLC.). Ramachandran plot analysis showed that 98.48%, 98.46%, 98.86%, and 98.02% residues of Atg40, FAM134B, Sec62, and RTN3 structures were in the favored regions, whereas no residues were in the outlier region. Parameters of crystallographic refinement are summarized in Supplementary Table 5.

### LigPlot + diagrams

2D protein interaction diagrams for Atg40^237–252^-Atg8, FAM134B^450–468^-GABARAP, SEC62^361–376^-GABARAP, and RTN3^245–264^ were generated by LigPlot+ ver. 2.1^51^. Hydrogen-bond calculation parameters were set to 2.70 and 3.35 as maximum H–A and D-A distances, respectively. Non-bonded contact parameters were set to 2.90 and 4.00 as minimum and maximum contact distances, respectively. Representative-hydrophobic-only option was applied for clarity.

### Isothermal titration calorimetry

Peptides used in ITC are listed in Supplementary Table 4. ITC experiments were performed using Microcal iTC200 calorimeter (Malvern Panalytical), with stirring at 1000 rpm at 25 °C. Atg40, FAM134B, SEC62, and RTN3 peptides were prepared as injection samples from the syringe. Concentrations of FAM134B, SEC62, and RTN3 peptides were 0.5 mM, whereas those of Atg40^234–252^ and Atg40^237–252^ peptides were 2.0 and 1.8 mM, respectively. In total, 200 μl Atg8, LC3A, LC3B, LC3C, GABARAP, GABARAPL1, and GABARAPL2 with 10 times lower concentration than the syringe samples were filled in the cell. As an exception, 1.8 mM Atg8 was filled in the cell when 1.8 mM Atg40^237–252^ D243A D247A was in a syringe. The titration involved 18 injections of 2 μl of the syringe sample at intervals of 120 s into a cell after one injection of 0.4 μl of syringe sample. Data sets obtained from the titration of the same syringe sample to the cell filled with buffer were used as reference data for subtraction of heat of dilution. MicroCal Origin 7.0 software was used for data analysis. Thermal measurement of the first injection of the syringe samples was removed from the analysis. Thermal titration data were fit to a single-site binding model, which determines thermodynamic parameter the enthalpy (ΔH), dissociation constant (Kd), and stoichiometry of binding (N). When the fitting was not convergent due to weak interaction, N was fixed to 1.0. The error of each parameter shows the fitting error.

### SNAP labeling

SNAP-GST-40C WT and Y242A M245A were labeled with SNAP-Surface Alexa Fluor 488 (New England Biolabs) according to the procedure. Briefly, 200 μM proteins were incubated with 20 μM SNAP-Surface Alexa Fluor 488, 1 mM DTT, 20 mM HEPES pH 6.8, and 150 mM NaCl for 10 min in a light-blocked box at room temperature. The labeled samples were used immediately and prepared at time of uses.

### Microscopic observation of phase separated droplets

Microscopic observation of phase separated droplets was performed as described previously^52^. Briefly, 10 μM mCherry-Atg8 or mCherry-Atg8-CC^tetramer^ was mixed with 10 μM SNAP-GST-40C WT or SNAP-GST-40C Y242A M245A. The samples were transferred to glass-bottom dishes (MatTek) with moisten paper for preventing sample evaporation. The samples were observed using FV3000RS confocal laser microscope (Olympus) equipped with ×60 objective lens (Olympus). To detect SNAP-Alexa 488 and mCherry fluorescence, the signals from 500 to 540 nm excited by 488-nm laser and from 570 to 670 nm excited by 561-nm laser were detected, respectively. For FRAP experiments, the ROI in the samples were photobleached for 0.542 s by 488-nm laser with 80% output intensity and recorded every 2.17 s for 215 s. At quantification, the fluorescence intensities were calculated by setting the value before and after the photobleaching to be 100 and 0%, respectively. To normalize ROI fluorescence, background fluorescence value was subtracted.

### SEC-MALS analysis

SEC-MALS was performed with the HPLC system (Shimadzu) equipped with a Shimadzu LC-20AD pump, a UV detector (SDP-20A), a MALS detector (DAWN HELEOS II, Wyatt Technology), and a differential refractive index (RI) detector (RI-501, Shodex). The sample was applied to Superdex 200 increase 10/300 column (GE Healthcare) equilibrated in buffer J at room temperature. The data analysis was carried out using Wyatt ASTRA software using a dn/dc value (protein 0.1853, DDM 0.1435) and UV Ext. coefficient (purified Atg40 1.220).

### Reporting summary

Further information on research design is available in the Nature Research Reporting Summary linked to this article.

## Supplementary information

Supplementary Information

Supplementary Movie 1

Reporting Summary

Description of Additional Supplementary Files

## Data Availability

The data supporting the finding of this study are available from the authors upon reasonable request. Source data for blots (Figs. 1a, 2b, 2c, 4b, 5c, Supplementary Figs. 1a, 1d, 2a, 6c, e, f) and graphs (Figs. 2d, 2e, 3, 4a, 4c-f, Supplementary Figs. 1b, 3, 4, 5b) are provided as a Source Data file. Coordinates and structure factors for Atg40-Atg8, FAM134B-GABARAP, SEC62-GABARAP, and RTN3-GABARAP have been deposited in the Protein Data Bank under accession codes 7BRN, 7BRQ, 7BRT, and 7BRU, respectively.

## Source data

Source data

## Code availability

ImageJ Scripts used in this study are provided in Supplementary Information.
