# Supplementary material for: Super-assembly of ER-phagy receptor Atg40 induces local ER remodeling at contacts with forming autophagosomal membranes
Source: Nat Commun. 2020 Jul 3;11:3306. doi: 10.1038/s41467-020-17163-y (PMC7335187; doi:10.1038/s41467-020-17163-y)
Supplement: Supplementary file 1 — Supplementary Information [file 41467_2020_17163_MOESM1_ESM.pdf]

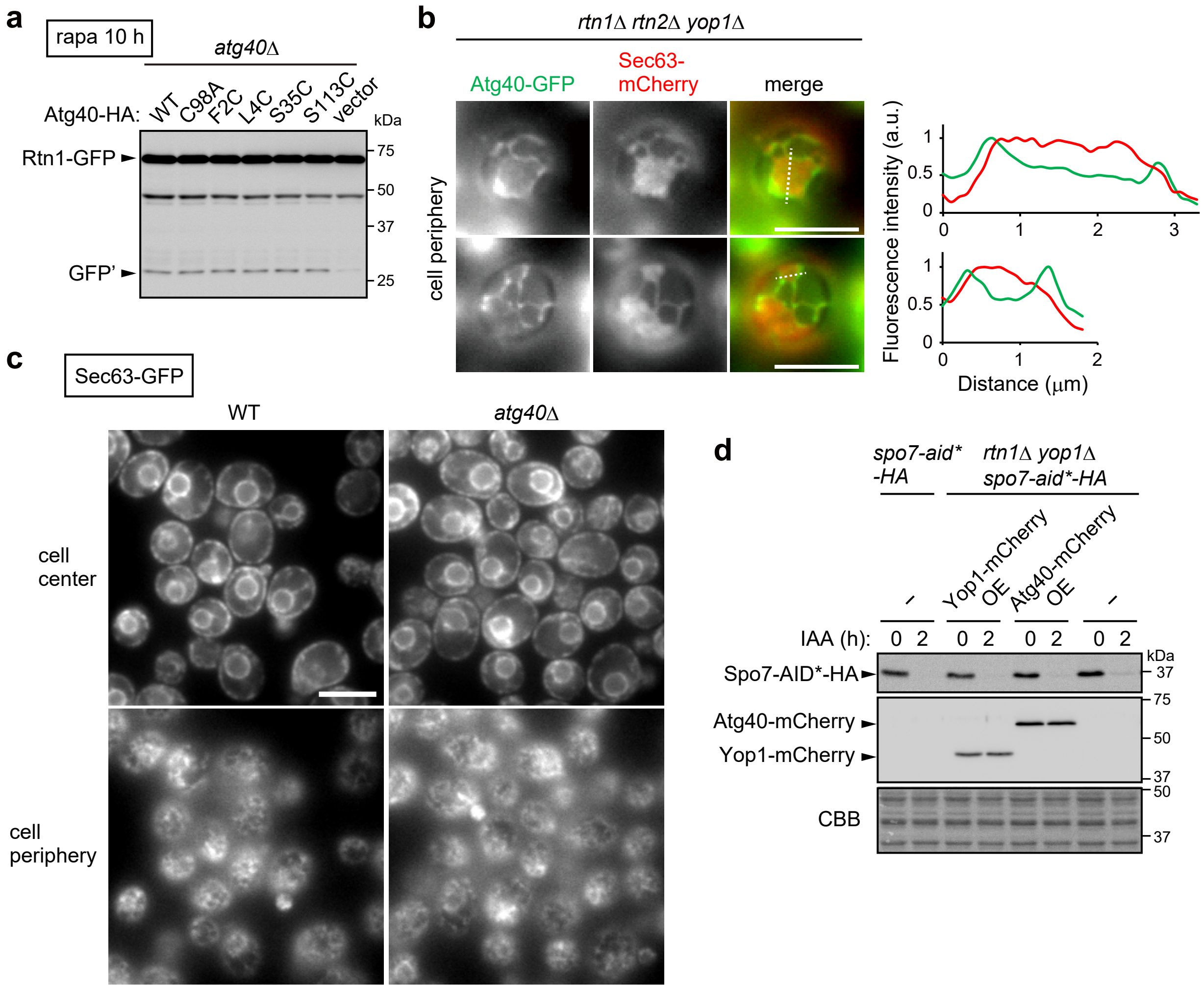

Supplementary Fig. 1. Atg40 is a reticulon-like protein that is dispensable for maintenance of overall ER structure.
a. Degradation of Rtn1-GFP in cells expressing Atg40-HA variants was measured by immunoblotting using an antibody against GFP. The amounts of GFP fragments (GFP'), which are generated by vacuolar cleavage of Rtn1-GFP, correlate with ER-phagy activity of the cells.
b. ER structure and localization of Atg40 in *rtn1*Δ *rtn2*Δ *yop1*Δ cells were analyzed by fluorescence microscopy of the cell periphery, showing that Atg40-GFP localized to the ER tubules and the edge of the ER sheets. Line scan analysis of dashed line shows the normalized fluorescence intensity.
c. ER morphology in *atg40*Δ cells was analyzed by fluorescence microscopy.
d. IAA-induced degradation of Spo7-AID*-HA was confirmed by immunoblotting using anti-HA antibody. CBB-stained membrane served as a loading control.
The experiments were repeated independently twice (a, b, d) or three times (c). Scale bars, 5 μm.

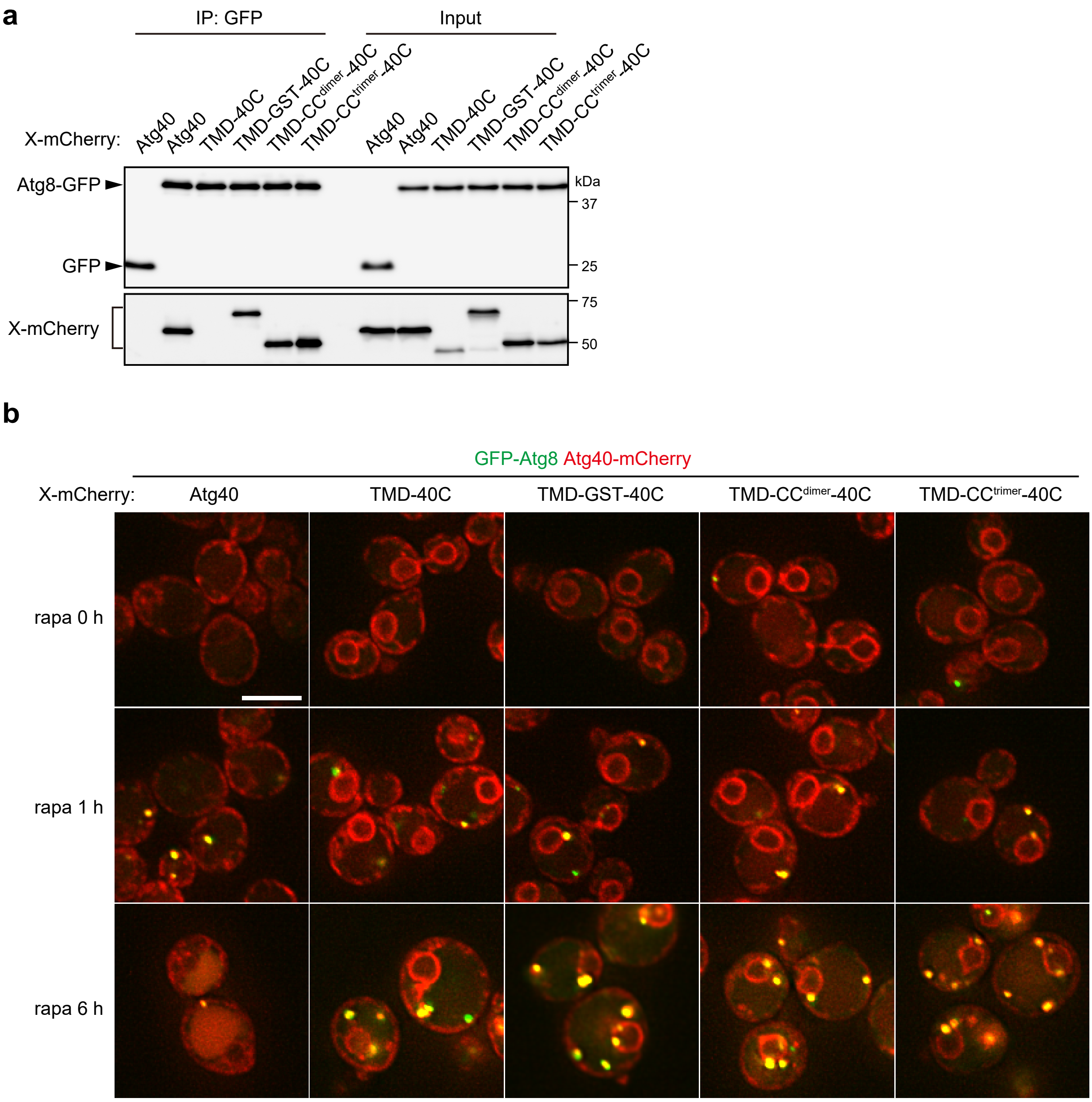

Supplementary Fig. 2. Non–reticulon-like proteins fused with the Atg40 C-terminal region form puncta depending on rapamycin treatment.

a. *ATG8-GFP atg4Δ atg40Δ* cells constitutively expressing the mCherry-tagged fusion proteins were treated with rapamycin for 6 h. Cell lysates (input) were subjected to immunoprecipitation using anti-GFP antibody, and immunoprecipitates (IP) were analyzed by immunoblotting using antibodies against GFP and mCherry.

b. *atg40Δ* cells constitutively expressing the mCherry-tagged fusion proteins were treated with rapamycin and analyzed by fluorescence microscopy. The images are maximum-intensity projections of Z-stacks (7 plane stacks, 0.2 µm spacing). The quantification results are shown in Fig 2d. Scale bars, 5 µm.

The experiments were repeated independently twice (a) or three times (b).

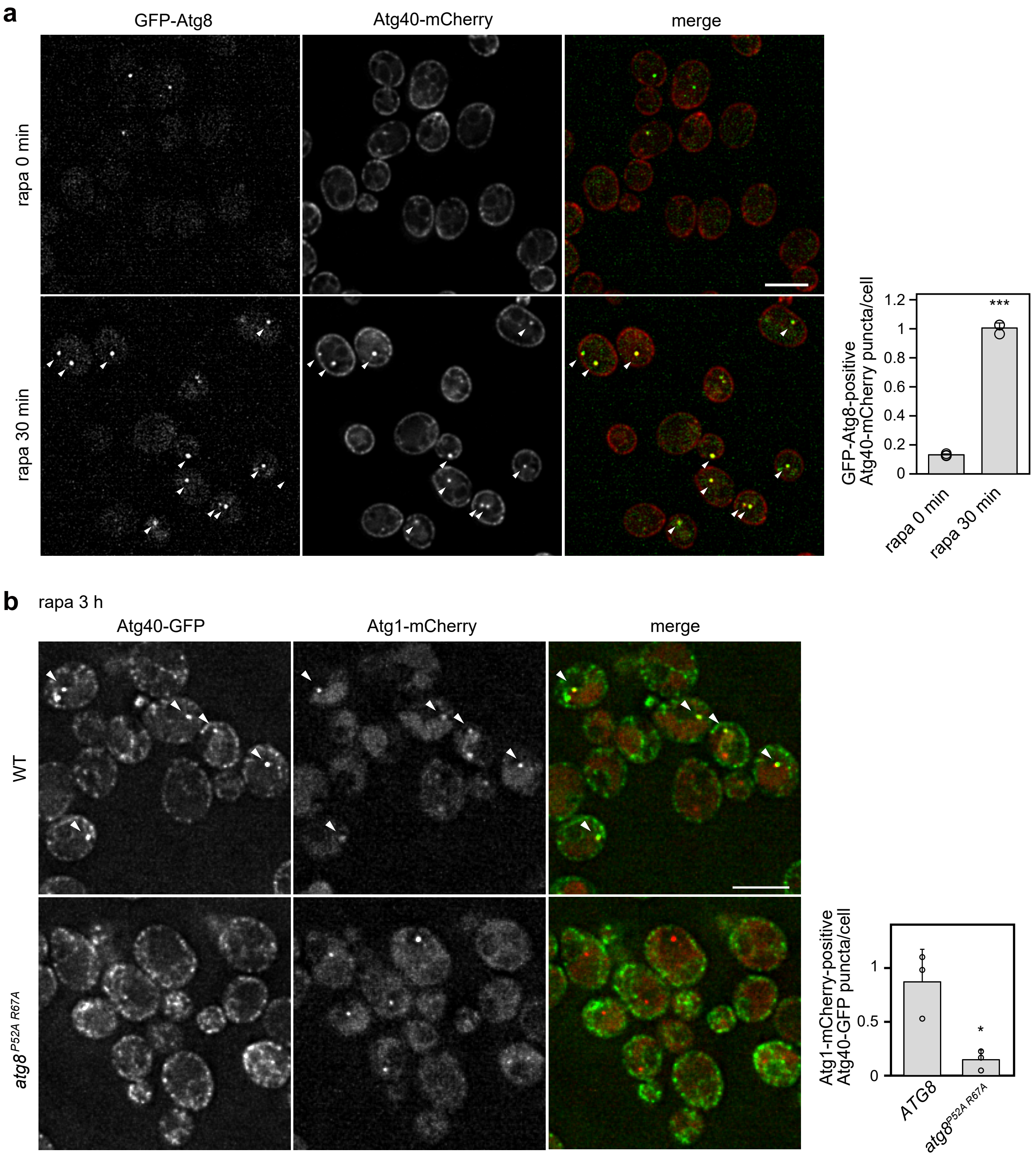

Supplementary Fig. 3. Atg40 assembly depends on rapamycin treatment and the binding to Atg8.

a. *GFP-ATG8* cells expressing Atg40-mCherry under the control of the *ADH* promoter were treated with rapamycin and analyzed by fluorescence microscopy. Arrowheads, GFP-Atg8-positive Atg40-mCherry puncta.

b. Cells coexpressing Atg40-GFP and Atg1-mCherry were treated with rapamycin and analyzed by fluorescence microscopy. The Atg8$^{P52A\ R67A}$ mutant is defective in the interaction with Atg40. Arrowheads, Atg1-mCherry-positive Atg40-GFP puncta.

The quantification results are shown as means ± s.d. (n=3). ****P* = 1.97 ×10^{-6} (a), **P* = 0.0164 (b) (unpaired two-tailed Student's *t*-test). Scale bars, 5 μm.

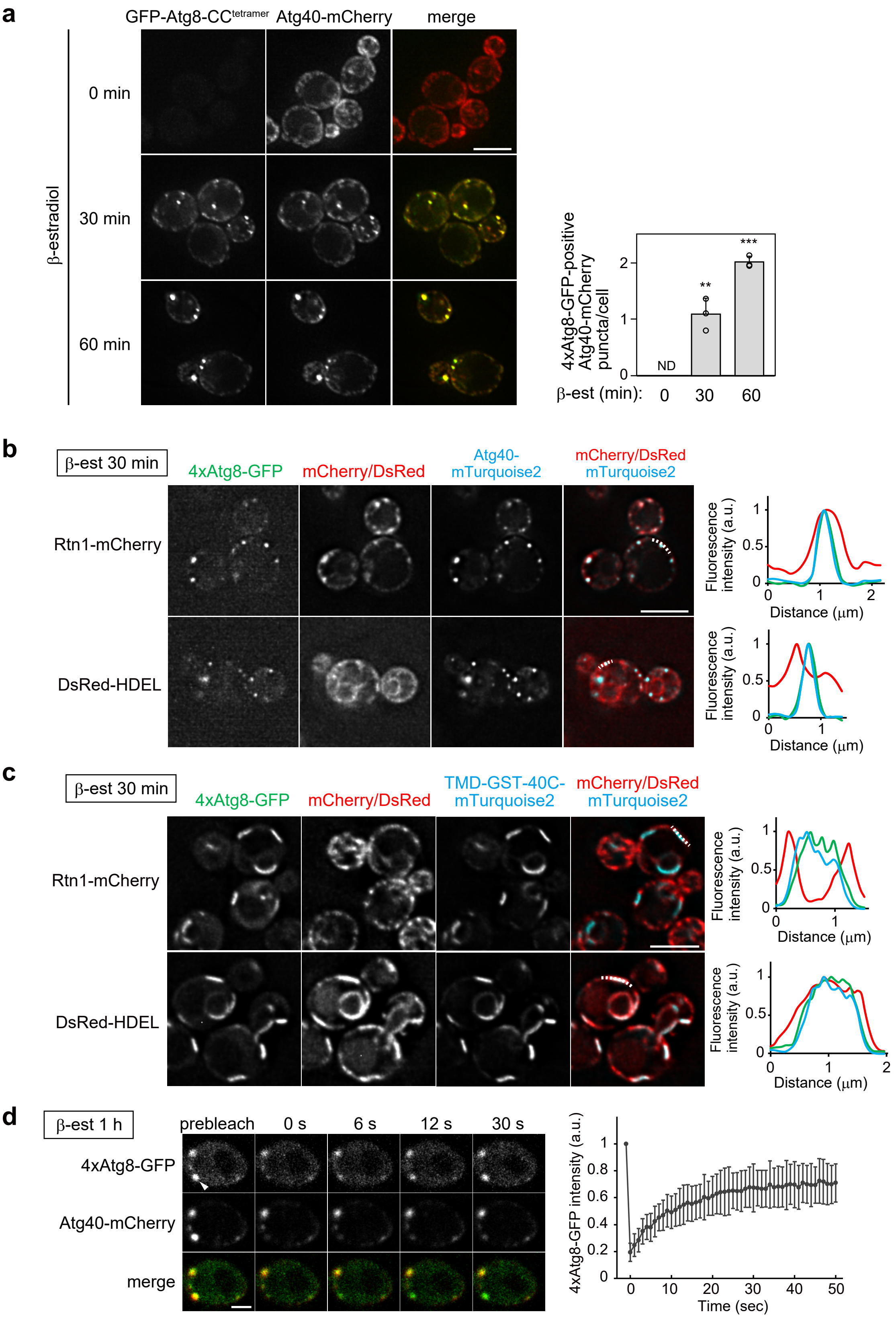

a
GFP-Atg8-CCtetramer
Atg40-mCherry
merge
β-estradiol
0 min
30 min
60 min
4xAtg8-GFP-positive Atg40-mCherry puncta/cell
ND
**
***
β-est (min): 0 30 60
b
β-est 30 min
4xAtg8-GFP
mCherry/DsRed
Atg40-mTurquoise2
mCherry/DsRed mTurquoise2
Rtn1-mCherry
DsRed-HDEL
Fluorescence intensity (a.u.)
Distance (μm)
c
β-est 30 min
4xAtg8-GFP
mCherry/DsRed
TMD-GST-40C-mTurquoise2
mCherry/DsRed mTurquoise2
Rtn1-mCherry
DsRed-HDEL
Fluorescence intensity (a.u.)
Distance (μm)
d
β-est 1 h
prebleach
0 s
6 s
12 s
30 s
4xAtg8-GFP
Atg40-mCherry
merge
4xAtg8-GFP intensity (a.u.)
Time (sec)

Supplementary Fig. 4. Characterization of 4×Atg8-induced Atg40 assemblies.

a. Cells coexpressing Atg40-mCherry and GFP-Atg8-CC^{tetramer} under the constitutive *ADH* promoter and the β-estradiol-inducible promoter, respectively, were analyzed by fluorescence microscopy. β-estradiol treatment induced expression of GFP-Atg8-CC^{tetramer} and clustering of Atg40-mCherry. The quantification results are shown as means ± s.d. (n=3). $^{**}P = 0.00259$ (30 min), $^{***}P = 4.53 \times 10^{-6}$ (60 min) (unpaired two-tailed Student's *t*-test).

b–d. Cells were treated with β-estradiol for 30 min (b, c) or 1 h (d) to induce 4×Atg8-GFP expression, and then analyzed by fluorescence microscopy. Line scan analysis of dashed line shows the normalized fluorescence intensity (b, c). In (d), images before and after photobleaching the 4×Atg8-GFP fluorescence at the indicated region (arrowheads) are shown. The normalized fluorescence intensity of the bleached regions was quantified and shown as means ± s.d. (n=13).

Scale bars, 5 μm (a-c), 2 μm (d).

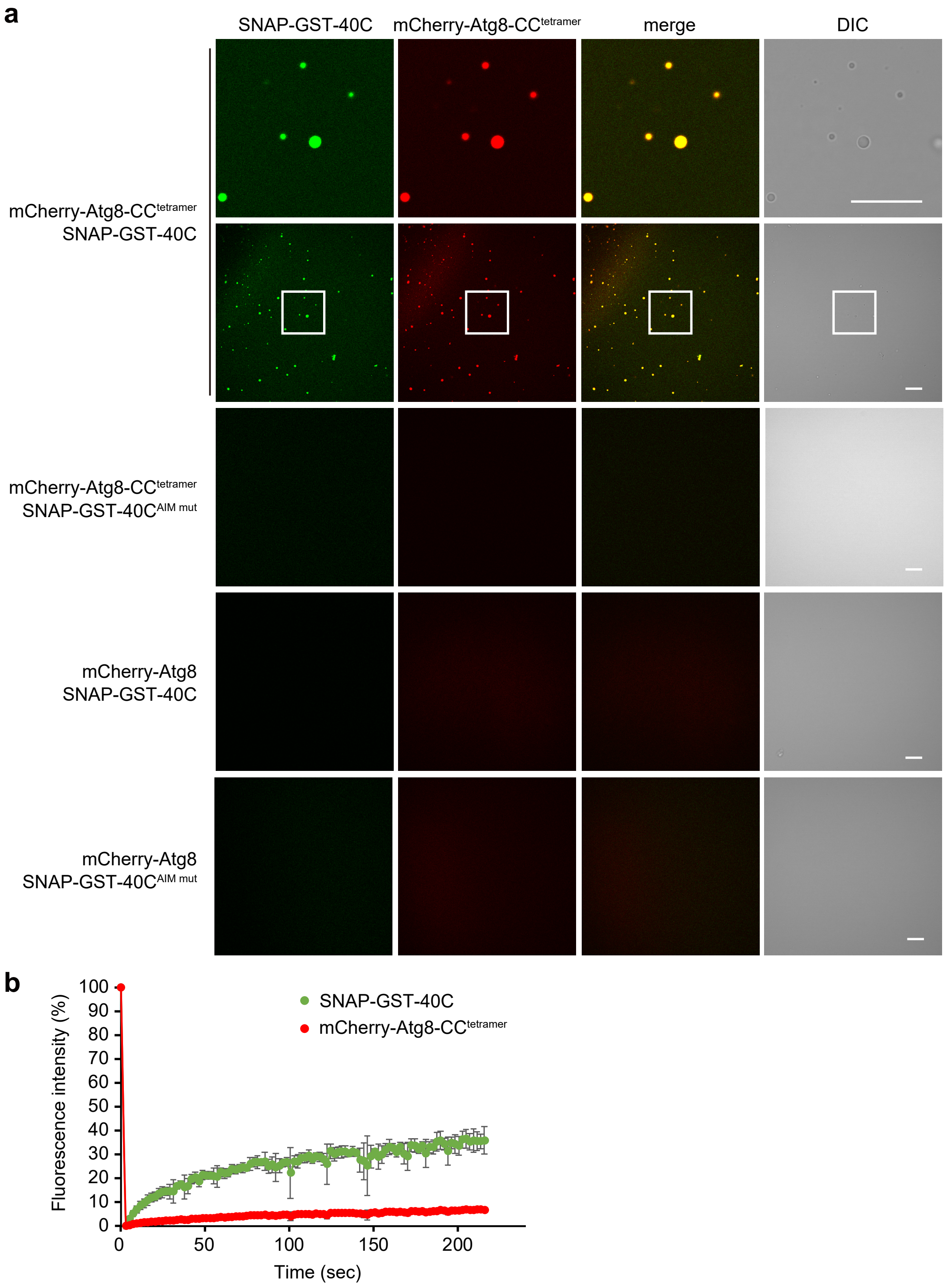

Supplementary Fig. 5. Multivalent interaction between tetramerized Atg8 and the dimerized C-terminal segment of Atg40 induces the formation of phase-separated droplets.

a. 10 μM mCherry-Atg8 or mCherry-Atg8-CC^{tetramer} was mixed with 10 μM SNAP-GST-40C or SNAP-GST-40C^{AIMmut} labeled with Alexa Fluor 488 and analyzed by confocal microscopy. The experiments were repeated independently twice. Scale bars, 20 μm.

b. FRAP analysis was performed on the protein condensates of mCherry-Atg8-CC^{tetramer} and SNAP-GST-40C in (a). Relative fluorescence intensity at the photobleached region was measured and the quantification results are shown as means ± s.d. (n=4).

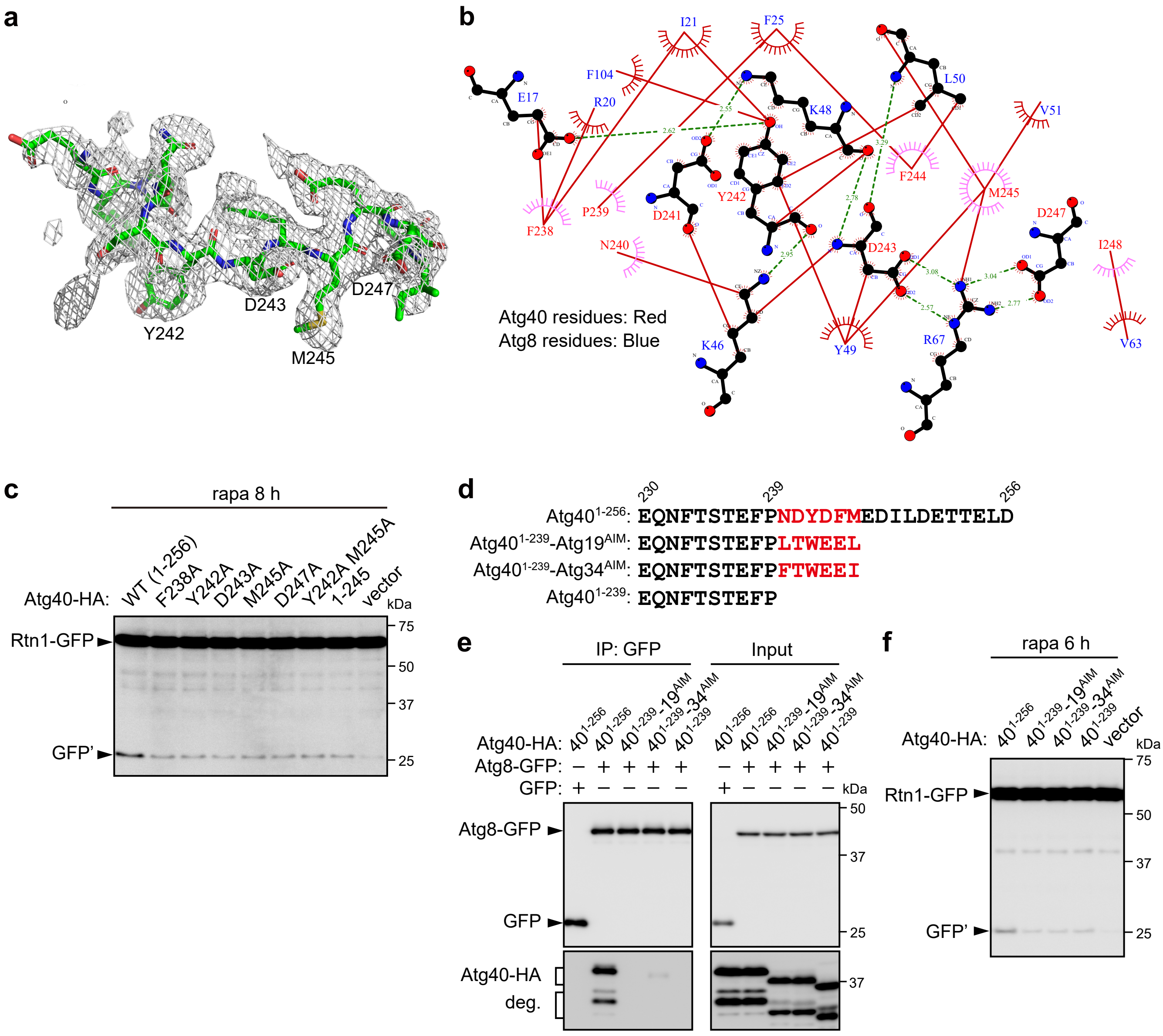

Supplementary Fig. 6. Atg8 binding of Atg40, enhanced by accessory interactions, is important for ER-phagy.

a. An unbiased mF_o-DF_c electron density map with Atg40 AIM (Atg40$^{237\text{-}249}$) contoured at 3 σ.

b. A two-dimensional protein interaction diagram for Atg40$^{237\text{-}252}$-Atg8.

c, f. ER-phagy activity in *atg40*Δ cells expressing the Atg40 mutants was assessed by monitoring vacuolar cleavage of Rtn1-GFP.

d. Amino acid sequences of the C-terminal region of Atg40 and the chimera proteins with the AIMs of Atg19 and Atg34 (Atg40$^{1\text{-}239}$-Atg19^{AIM} and Atg40$^{1\text{-}239}$-Atg19^{AIM}), respectively.

e. *ATG8-GFP atg4*Δ *atg40*Δ cells expressing Atg40-HA were treated with rapamycin for 6 h, and subjected to immunoprecipitation analysis as described in Fig. 5c.

The experiments were repeated independently twice (c, e, f).

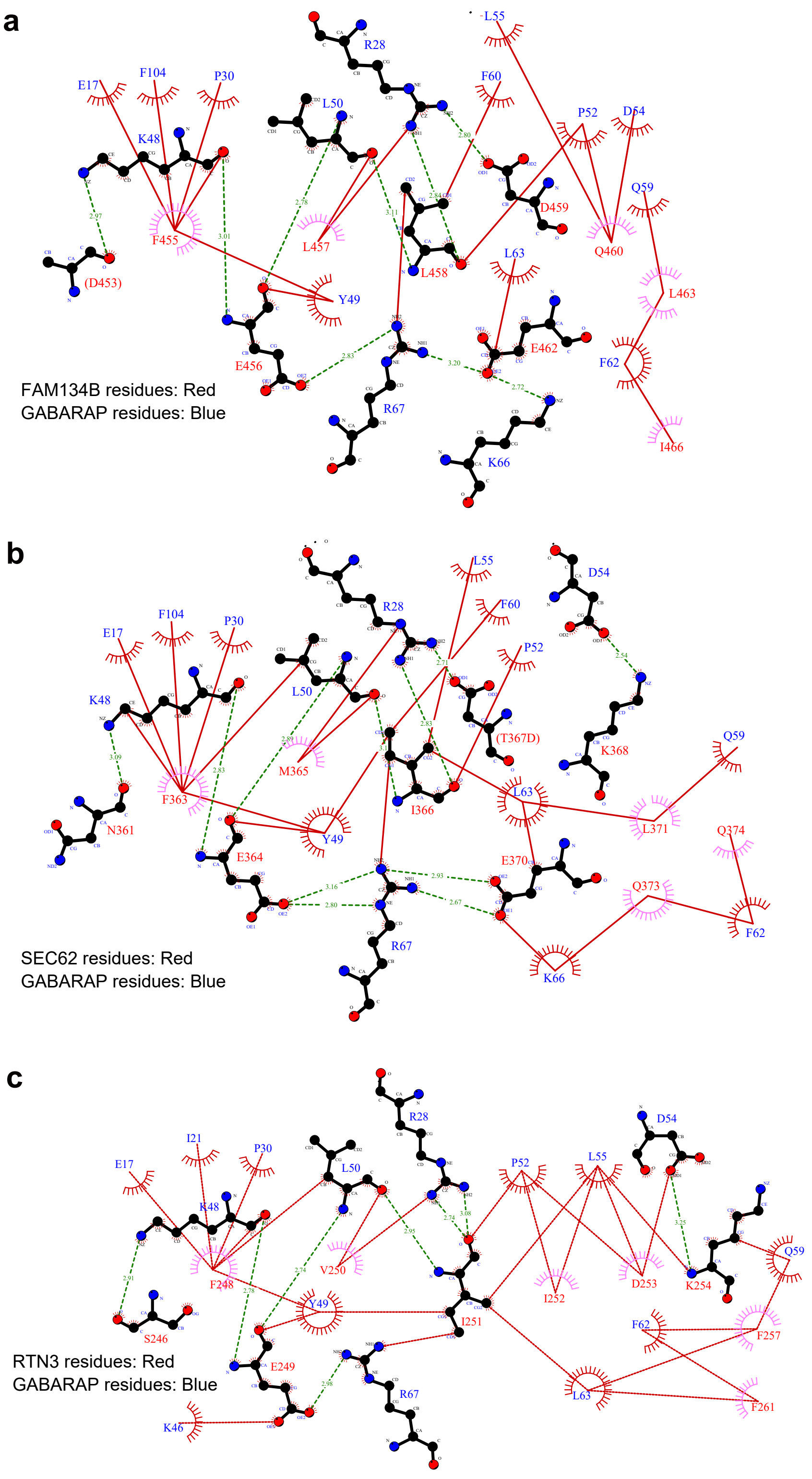

Supplementary Fig. 7. Interactions in FAM134B$^{450\text{-}468}$-GABARAP, SEC62$^{361\text{-}376}$-GABARAP, and RTN3$^{245\text{-}264}$-GABARAP

a-c. Two-dimensional protein–protein interaction diagrams for FAM134B$^{450\text{-}468}$-GABARAP (a), SEC62$^{361\text{-}376}$-GABARAP (b), and RTN3$^{245\text{-}264}$-GABARAP (c).

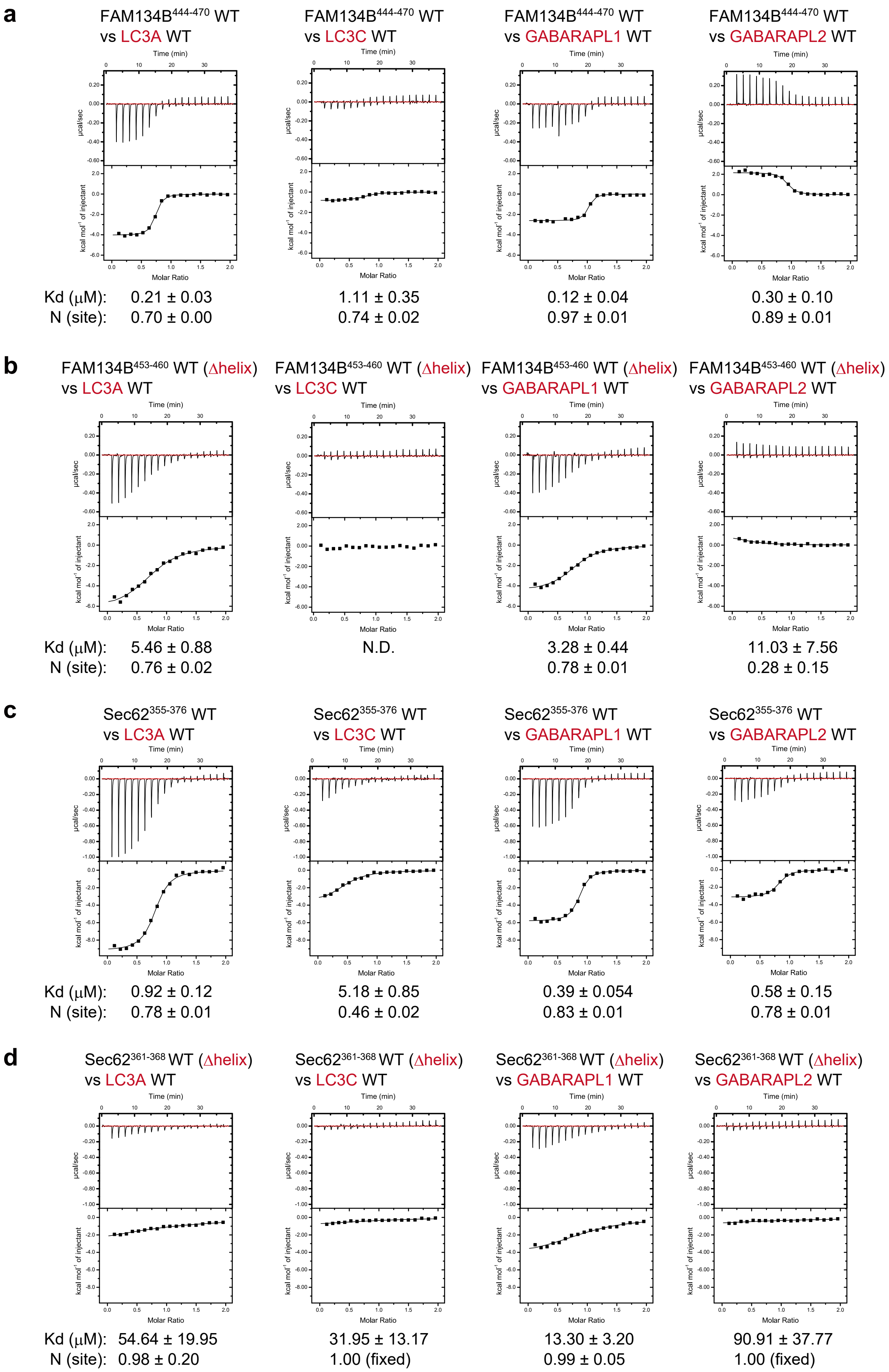

a
FAM134B444-470 WT vs LC3A WT
FAM134B444-470 WT vs LC3C WT
FAM134B444-470 WT vs GABARAPL1 WT
FAM134B444-470 WT vs GABARAPL2 WT
Time (min)
µcal/sec
kcal mol-1 of injectant
Molar Ratio
Kd (µM): 0.21 ± 0.03 | 1.11 ± 0.35 | 0.12 ± 0.04 | 0.30 ± 0.10
N (site): 0.70 ± 0.00 | 0.74 ± 0.02 | 0.97 ± 0.01 | 0.89 ± 0.01
b
FAM134B453-460 WT (Δhelix) vs LC3A WT
FAM134B453-460 WT (Δhelix) vs LC3C WT
FAM134B453-460 WT (Δhelix) vs GABARAPL1 WT
FAM134B453-460 WT (Δhelix) vs GABARAPL2 WT
Kd (µM): 5.46 ± 0.88 | N.D. | 3.28 ± 0.44 | 11.03 ± 7.56
N (site): 0.76 ± 0.02 | 0.78 ± 0.01 | 0.28 ± 0.15
c
Sec62355-376 WT vs LC3A WT
Sec62355-376 WT vs LC3C WT
Sec62355-376 WT vs GABARAPL1 WT
Sec62355-376 WT vs GABARAPL2 WT
Kd (µM): 0.92 ± 0.12 | 5.18 ± 0.85 | 0.39 ± 0.054 | 0.58 ± 0.15
N (site): 0.78 ± 0.01 | 0.46 ± 0.02 | 0.83 ± 0.01 | 0.78 ± 0.01
d
Sec62361-368 WT (Δhelix) vs LC3A WT
Sec62361-368 WT (Δhelix) vs LC3C WT
Sec62361-368 WT (Δhelix) vs GABARAPL1 WT
Sec62361-368 WT (Δhelix) vs GABARAPL2 WT
Kd (µM): 54.64 ± 19.95 | 31.95 ± 13.17 | 13.30 ± 3.20 | 90.91 ± 37.77
N (site): 0.98 ± 0.20 | 1.00 (fixed) | 0.99 ± 0.05 | 1.00 (fixed)

Supplementary Fig. 8. ITC analysis of the interactions of FAM134B and SEC62 peptides with mammalian Atg8 homologues.

a–d. The binding affinity of FAM134B$^{444\text{-}470}$ (a), FAM134B$^{453\text{-}460}$ (b), SEC62$^{355\text{-}376}$ (c), and SEC62$^{361\text{-}368}$ (d) peptides to mammalian Atg8 homologues was measured by ITC.

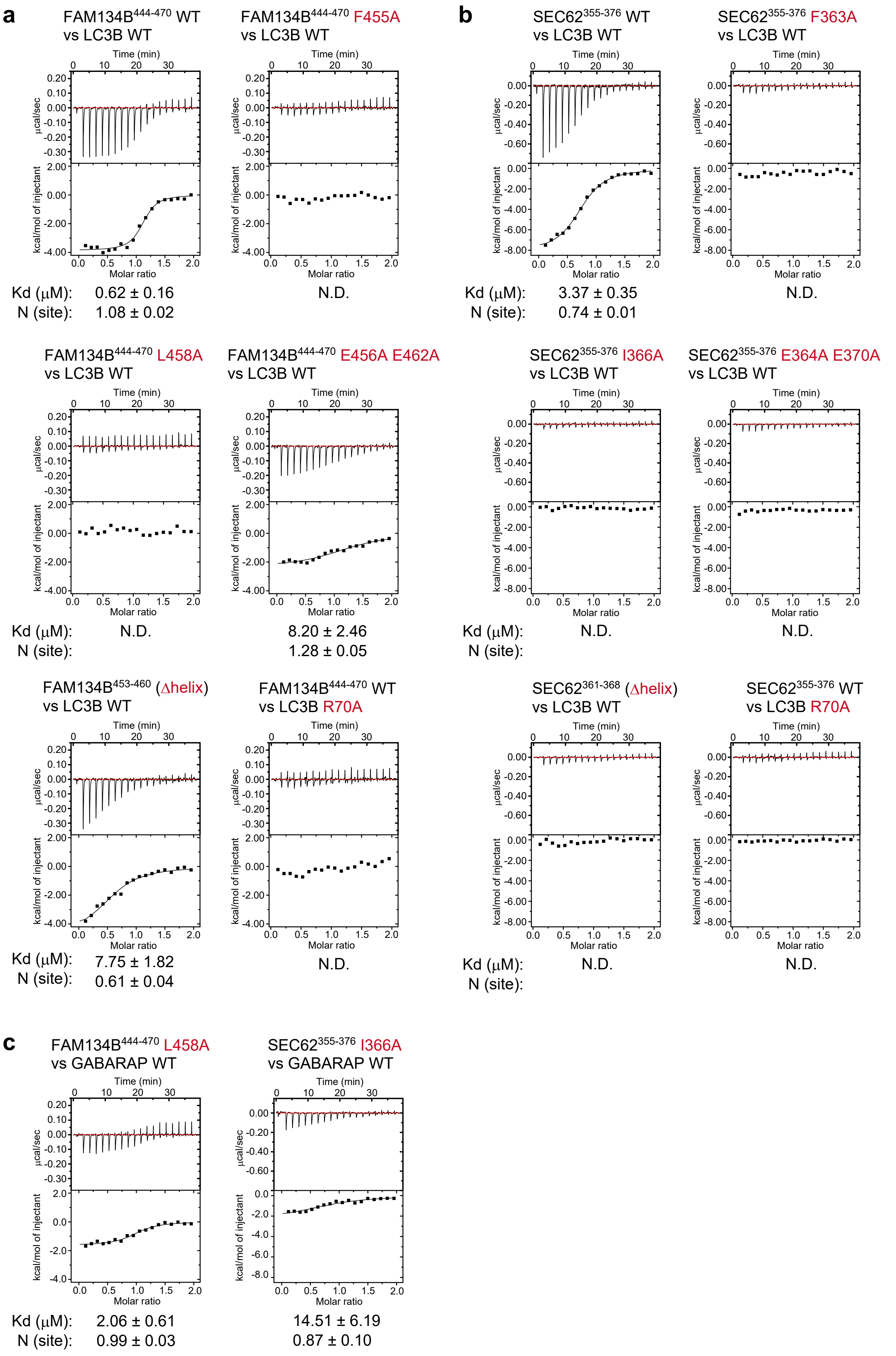

Supplementary Fig. 9. ITC analysis of the interactions of FAM134B and SEC62 peptides with GABARAP/LC3B. **a–c.** The binding affinity of FAM134B and SEC62 peptides to LC3B (a, b) or GABARAP (c) was measured by ITC.

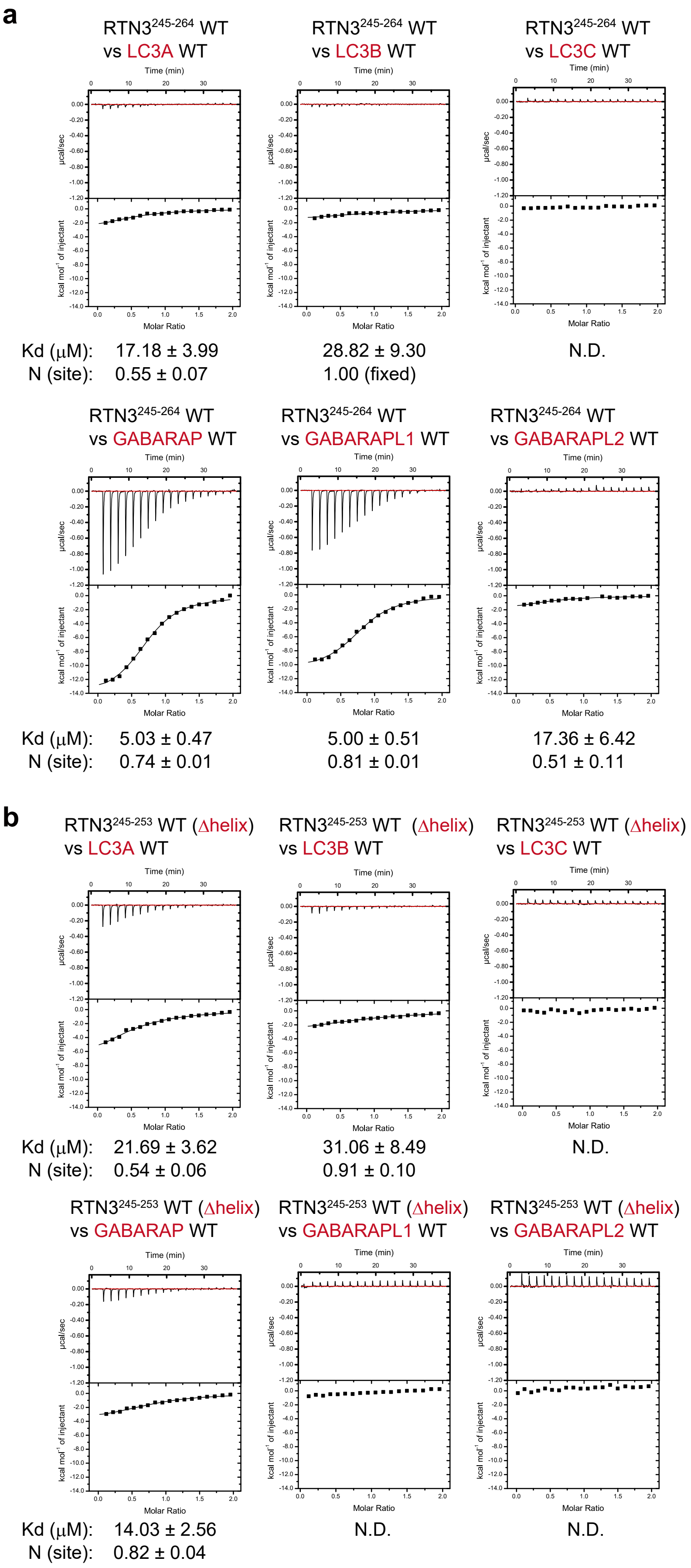

Supplementary Fig. 10. ITC analysis of the interactions of RTN3 peptides with mammalian Atg8 homologues. **a, b.** The binding affinity of RTN3$^{245\text{-}264}$ (a) and RTN3$^{245\text{-}253}$ (b) peptides to mammalian Atg8 homologues was measured by ITC.

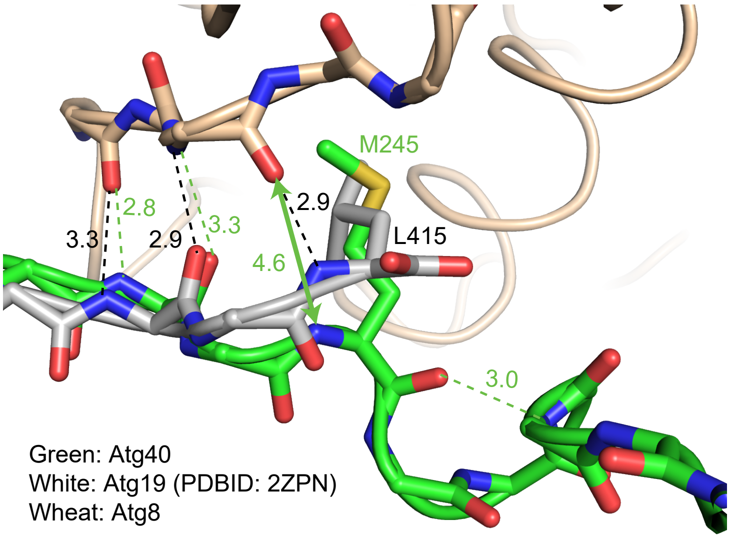

Supplementary Fig. 11. Comparing the structural configurations of the AIM-containing regions of Atg40 and Atg19 bound to Atg8.

The structure of the Atg19 region bound to Atg8 (PDB ID: 2ZPN) was superimposed with that of the Atg40 region in complex with Atg8.

Supplementary Table 1. Yeast strains used in this study

Name	Genotype	Background	Figure
YKM752	*atg8Δ::TRP1 atg4Δ::hphNT1 atg40Δ::natNT2*	BJ3505	1a
YKM770	*SEC63-EGFP-kanMX4*	BY4741	1b
YKM1079	*SEC63-EGFP-kanMX4 rtn1Δ::zeoNT3 rtn2Δ::hphNT1 yop1Δ::natNT2 atg40Δ::CgHIS3*	BY4741	1b
YKM1964	*ADE2 ura3::ADH1pro-OsTIR1-9Myc-URA3 spo7-aid*-3HA-hphNT1*	W303-1A	1c, Suppl 1d
YKM1965	*ADE2 ura3::ADH1pro-OsTIR1-9Myc-URA3 spo7-aid*-3HA-hphNT1 rtn1Δ::kanMX4 yop1Δ::zeoNT3*	W303-1A	1c, Suppl 1d
YKM1979	*ADE2 ura3::ADH1pro-OsTIR1-9Myc-URA3 spo7-aid*-3HA-hphNT1 rtn1Δ::kanMX4 yop1Δ::zeoNT3 his3::*pRS303-*GPDpro-YOP1-mCherry*	W303-1A	1c, Suppl 1d
YKM1980	*ADE2 ura3::ADH1pro-OsTIR1-9Myc-URA3 spo7-aid*-3HA-hphNT1 rtn1Δ::kanMX4 yop1Δ::zeoNT3 his3::*pRS303-*GPDpro-ATG40-mCherry*	W303-1A	1c, Suppl 1d
YKM939	*SEC63-EGFP-kanMX4 atg40Δ::natNT2*	BY4741	2b, c, Suppl 1c
YKM2064	*ADE2 leu2::GFPplus-ATG8-hphNT1 atg40Δ::zeoNT3 his3::*pRS303-*CUP1pro-ATG40-mCherry*	W303-1A	2d, Suppl 2b
YKM2066	*ADE2 leu2::GFPplus-ATG8-hphNT1 atg40Δ::zeoNT3 his3::*pRS303-*TEFpro-TMD-GST-40C-mCherry*	W303-1A	2d, Suppl 2b
YKM2067	*ADE2 leu2::GFPplus-ATG8-hphNT1 atg40Δ::zeoNT3 his3::*pRS303-*TEFpro-TMD-40C-CC^{dimer}-mCherry*	W303-1A	2d, Suppl 2b
YKM2068	*ADE2 leu2::GFPplus-ATG8-hphNT1 atg40Δ::zeoNT3 his3::*pRS303-*TEFpro-TMD-40C-CC^{trimer}-mCherry*	W303-1A	2d, Suppl 2b
YKM2069	*ADE2 leu2::GFPplus-ATG8-hphNT1 atg40Δ::zeoNT3 his3::*pRS303-*TEFpro-TMD-40C-mCherry*	W303-1A	2d, Suppl 2b
YKM1931	*ADE2 leu2::GFPplus-ATG8-hphNT1 his3::DsRed-HDEL-HIS3 atg40Δ::zeoNT3*	W303-1A	2e
YKM1560	*ADE2 ATG40-EGFP-kanMX6 ypt7Δ::mCherry-ATG8 (G116)-zeoNT3*	W303-1A	3a
YKM2076	*ADE2 ATG40-EGFP-kanMX6 ypt7Δ::mCherry-ATG8 (G116)-zeoNT3 atg1Δ::hphNT1*	W303-1A	3a
YKM1470	*ATG40-EGFP-kanMX6 ATG1-2xmCherry-hphNT1*	BY4741	3b, c

YKM1562	*ATG40-EGFP-kanMX6 ATG1-2xmCherry-hphNT1 atg2Δ::zeoNT3*	BY4741	3b
YKM1564	*ATG40-EGFP-kanMX6 ATG1-2xmCherry-hphNT1 atg8Δ::zeoNT3*	BY4741	3b
YKM1581	*ATG40-EGFP-kanMX6 ATG1-2xmCherry-hphNT1 atg14Δ::zeoNT3*	BY4741	3b
YKM1807	*atg40(Y242A M245A)-EGFP-kanMX6 ATG1-2xmCherry-hphNT1*	BY4741	3c
YKM1387	*kanMX4-ADHpro(truncated)-ATG40-mCherry-hphNT1 his3::GFP-ATG8 (G116)-zeoNT3*	BY4741	3d
YKM792	*ATG40-TEV-3FLAG-kan*	BJ3505	4b
YKM1709	*ATG40-TEV-3FLAG-kan atg1Δ::zeoNT3*	BJ3505	4b
YKM1670	*ATG40-TEV-3FLAG-kan atg2Δ::zeoNT3*	BJ3505	4b
YKM1657	*ATG40-TEV-3FLAG-kan atg8Δ::zeoNT3*	BJ3505	4b
YKM1674	*atg40 (Y242A M245A)-TEV-3FLAG-kan*	BJ3505	4b
YKM1868	*ADE2 leu2::pACT1-Z4EV-NAT-leu2d0-int kanMX4-ADHpro(truncated)-ATG40-mCherry-zeoNT3 ura3*::pRS306-*Z4EVpro-4xATG8 (G116A)-EGFP*	W303-1A	4c
YKM1869	*ADE2 leu2::pACT1-Z4EV-NAT-leu2d0-int kanMX4-ADHpro(truncated)-atg40 (Y242A M245A)-mCherry-zeoNT3 ura3*::pRS306-*Z4EVpro-4xATG8 (G116A)-EGFP*	W303-1A	4c
YKM1988	*ADE2 leu2::pACT1-Z4EV-NAT-leu2d0-int atg40Δ::zeoNT3 ura3*::pRS306-*Z4EVpro-4xATG8(G116A)-EGFP YOP1-2xmCherry-hphNT1 his3*::pRS303-*ADHpro-ATG40-mTurquoise2*	W303-1A	4d
YKM2062	*ADE2 leu2::pACT1-Z4EV-NAT-leu2d0-int atg40Δ::zeoNT3 ura3::pRS306-Z4EVpro-4xATG8 (G116A)-EGFP his3*::pRS303-*TEFpro-TMD-GST-40C-mTurquoise2 YOP1-2xmCherry-hphNT1*	W303-1A	4e
YKM1948	*ADE2 ypt7Δ:2xmTagBFP2-ATG8-hphNT1 YOP1-2xmNeonGreen-zeoNT3 his3*::pRS303-*ADHpro (truncated)-ATG40-mCherry*	W303-1A	4f
YKM1978	*ADE2 ypt7Δ:2xmTagBFP2-ATG8-hphNT1 his3*::pRS303-*ADHpro (truncated)-ATG40-mCherry ura3::pRS306-ADHpro-GFP-HDEL*	W303-1A	4f
YKM1638	*ATG8-EGFP-kanMX6 atg4Δ::hphNT1 atg40Δ::natNT2*	BJ3505	5c, Suppl 2a, 6e
YKM853	*RTN1-EGFP-kanMX4 atg40Δ::natNT2*	BY4741	Suppl 1a, 6c, 6f
YKM777	*SEC63-mCherry-kan rtn1Δ::zeoNT3 rtn2Δ::hphNT1 yop1Δ::natNT2 ATG40-EGFP-HIS3MX6*	BY4741	Suppl 1b
YKM936	*SEC63-EGFP-kanMX4*	BY4741	Suppl 1c

YKM1653	*atg8*Δ::*EGFP-kanMX6 atg4*Δ::*hphNT1 atg40*Δ::*natNT2*	BJ3505	Suppl 2a, 6e
YKM1475	*kanMX4-ADHpro (truncated)-ATG40-mCherry-hphNT1 his3*::*GFP-ATG8 (G116)-zeoNT3 YOP1-2xmTagBFP2-HIS3MX6*	BY4741	Suppl 3a
YKM1153	*ATG8 wt-T-P-kanMX ATG40-EGFP-HIS3MX6 ATG1-2xmCherry-hphNT1*	BY4741	Suppl 3b
YKM1154	*atg8 (P52A R67A)-T-P-kanMX ATG40-EGFP-HIS3MX6 ATG1-2xmCherry-hphNT1*	BY4741	Suppl 3b
YKM2086	*ADE2 leu2*::*pACT1-Z4EV-NAT-leu2d0-int kanMX4-ADHpro (truncated)-ATG40-mCherry-hphNT1 ura3*::pRS306-*Z4EVpro-EGFP-Atg8 (K26P)-CC*^{tetramer} *atg4*Δ::*zeoNT3*	W303-1A	Suppl 4a
YKM1986	*ADE2 leu2*::*pACT1-Z4EV-NAT-leu2d0-int atg40*Δ::*zeoNT3 ura3*::pRS306-*Z4EVpro-4xATG8 (G116A)-EGFP RTN1-2xmCherry-hphNT1 his3*::pRS303-*ADHpro-ATG40-mTurquoise2*	W303-1A	Suppl 4b
YKM1987	*ADE2 leu2*::*pACT1-Z4EV-NAT-leu2d0-int atg40*Δ::*zeoNT3 ura3*::pRS306-*Z4EVpro-4xATG8 (G116A)-EGFP trp1*::*DsRed-Express2-HDEL his3*::pRS303-*ADHpro-ATG40-mTurquoise2*	W303-1A	Suppl 4b
YKM2060	*ADE2 leu2*::*pACT1-Z4EV-NAT-leu2d0-int atg40*Δ::*zeoNT3 ura3*::pRS306-*Z4EVpro-4xATG8 (G116A)-EGFP his3*::pRS303-*TEFpro-TMD-GST-40C-mTurquoise2 RTN1-2xmCherry-hphNT1*	W303-1A	Suppl 4c
YKM2063	*ADE2 leu2*::*pACT1-Z4EV-NAT-leu2d0-int atg40*Δ::*zeoNT3 ura3*::pRS306-*Z4EVpro-4xATG8 (G116A)-EGFP his3*::pRS303-*TEFpro-TMD-GST-40C-mTurquoise2 trp1*::*DsRed-Express2-HDEL*	W303-1A	Suppl 4c

Supplementary Table 2. Plasmids used in this study

Name	Description
pKE135	pRS426-*ATG40-6HA*
pKE182	pRS426-*ATG40 (C98A)-6HA*
pKE183	pRS426-*ATG40 (F2C)-6HA*
pKE184	pRS426-*ATG40 (L4C)-6HA*
pKE185	pRS426-*ATG40 (S35C)-6HA*
pKE186	pRS426-*ATG40 (S113C)-6HA*
pKE255	pRS316-*CUP1pro-ATG40-mCherry*
pKE256	pRS316-*CUP1pro-YOP1-40C-mCherry*
pKE273	pRS316-*CUP1pro-YOP1-40C$^{AIM\ mut}$-mCherry*
pKE257	pRS316-*CUP1pro-YOP1-mCherry*
pKE258	pRS316-*CUP1pro-RTN1-40C-mCherry*
pKE274	pRS316-*CUP1pro-RTN1-40C$^{AIM\ mut}$-mCherry*
pKE259	pRS316-*CUP1pro-RTN1-mCherry*
pKE360	p416-*TEFpro-TMD-40C-mCherry*
pKE387	p416-*TEFpro-TMD-GST-40C-mCherry*
pKE389	p416-*TEFpro-TMD-CC^{dimer}-40C-mCherry*
pKE390	p416-*TEFpro-TMD-CC^{trimer}-40C-mCherry*
pKE422	pRS314-*TEFpro-TMD-GST-40C-mTagBFP2*
pKE92	pRS316-*ATG40*
pKE93	pRS316-*ATG40-EGFP*
pKE131	pRS316-*atg40 (Y242A M245A)-EGFP*
pKE108	pRS314-*ATG40-6HA*
pKE117	pRS314-*atg40 (Y242A M245A)-6HA*
pKE321	pRS316-*ATG40-6HA*
pKE342	pRS316-*atg40 (1-239)-6HA*
pKE343	pRS316-*atg40 (1-239)-LTWEEL-6HA*
pKE344	pRS316-*atg40 (1-239)-FTWEEI-6HA*
pKE424	pRS316-*atg40 (F238A)-6HA*
pKE425	pRS316-*atg40 (Y242A)-6HA*
pKE426	pRS316-*atg40 (D243A)-6HA*
pKE427	pRS316-*atg40 (M245A)-6HA*
pKE428	pRS316-*atg40 (1-245)-6HA*

pKE435	pRS316-*atg40 (D247A)-6HA*
pKE436	pRS316-*atg40 (Y242A M245A)-6HA*
-	pFA6a-*AID*-3HA-hphNT1*
pKE340	pRS303-*GPDpro-ATG40-mCherry*
pKE341	pRS303-*GPDpro-YOP1-mCherry*
pKE296	pRS303-*CUP1pro-ATG40-mCherry*
pKE537	pRS303-*TEFpro-TMD-GST-40C-mCherry*
pKE538	pRS303-*TEFpro-TMD-40C-*CC^{dimer}*-mCherry*
pKE539	pRS303-*TEFpro-TMD-40C-*CC^{trimer}*-mCherry*
pKE540	pRS303-*TEFpro-TMD-40C-mCherry*
pKE349	pRS306-*Z4EVpro-4xATG8-EGFP*
pKE496	pRS303-*ADHpro (truncated)-ATG40-mTurquoise2*
pKE536	pRS303-*TEFpro-TMD-GST-40C-mTurquoise2*
pKE212	pRS306-*ADHpro-GFP-HDEL*
pKE557	pRS306-*Z4EVpro-EGFP-Atg8 (K26P)-*$CC^{tetramer}$

Supplementary Table 3. Oligonucleotides used in this study

Name	Sequence
ATG40-C98A-Fw	tcgtttggaagtattGCTttgacaagagagctg
ATG40-C98A-Rv	cagctctcttgtcaaAGCaatacttccaaacga
ATG40-F2C-Fw	catagaaaactaatgTGTaatttaattttatgg
ATG40-F2C-Rv	ccataaaattaaattACAcattagttttctatg
ATG40-L4C-Fw	aaactaatgtttaatTGTattttatggccctta
ATG40-L4C-Rv	taagggccataaaatACAattaaacattagttt
ATG40-S35C-Fw	tcttccgttgacttcTGTaaggcaagtgctgct
ATG40-S35C-Rv	agcagcacttgccttACAgaagtcaacggaaga
ATG40-S113C-Fw	attgcctttatggaaTGTcaaaacaagcttacc
ATG40-S113C-Rv	ggtaagcttgttttgACAttccataaaggcaat
ATG40 (1-239)-Fw	ACCAGCACTGAGTTCCCGggtggtgcagcaggagga
ATG40 (1-239)-Rv	tcctcctgctgcaccaccCGGGAACTCAGTGCTGGT
ATG40 (1-239)-LTWEEL-Fw	accagcactgagttcccgCTGACTTGGGAAGAACTCggtggtgcagcaggagga
ATG40 (1-239)-LTWEEL-Rv	tcctcctgctgcaccaccGAGTTCTTCCCAAGTCAGcgggaactcagtgctggt
ATG40 (1-239)-FTWEEI-Fw	accagcactgagttcccgTTTACTTGGGAAGAAATAggtggtgcagcaggagga
ATG40 (1-239)-FTWEEI-Rv	tcctcctgctgcaccaccTATTTCTTCCCAAGTAAAcgggaactcagtgctggt
ATG40-F238A-Fw	caaaattttaccagcactgagGCTccgaatgattatgattttatg
ATG40-F238A-Rv	cataaaatcataatcattcggAGCctcagtgctggtaaaattttg
ATG40-Y242A-Fw	gagttcccgaatgatGCTgattttatggaggatattc
ATG40-Y242A-Rv	gaatatcctccataaaatcAGCatcattcgggaactc
ATG40-D243A-Fw	gttcccgaatgattatGCTtttatggaggatattctag
ATG40-D243A-Rv	ctagaatatcctccataaaAGCataatcattcgggaac
ATG40-M245A-Fw	cccgaatgattatgattttGCTgaggatattctagatgag
ATG40-M245A-Rv	ctcatctagaatatcctcAGCaaaatcataatcattcggg
ATG40-D247A-Fw	tatgattttatggagGCTattctagatgagaca
ATG40-D247A-Rv	tgtctcatctagaatAGCctccataaaatcata
ATG40-Y242A M245A-Fw	gagttcccgaatgatGCTgattttGCTgaggatattctagat
ATG40-Y242A M245A-Rv	atctagaatatcctcAGCaaaatcAGCatcattcgggaactc

ATG40 (1-245)-Fw	CCGAATGATTATGATTTTATGggtggtgcagcaggag
ATG40 (1-245)-Rv	ctcctgctgcaccaccCATAAAATCATAATCATTCGG

Supplementary Table 4. Peptides used in ITC experiments

Peptides	Production	Sequence
Atg40$^{237-252}$ WT	*E. coli*	GPEFPNDYDFMEDILDET
Atg40$^{237-252}$ M245A	Synthesis	EFPNDYDFAEDILDET
Atg40$^{237-252}$ D243A D247A	Synthesis	EFPNDYAFMEAILDET
Atg40$^{234-252}$ WT	Synthesis	YTSTEFPNDYDFMEDILDET
Atg40$^{234-252}$ F238A	Synthesis	YTSTEAPNDYDFMEDILDET
Atg40$^{234-252}$ Y242A	Synthesis	YTSTEFPNDADFMEDILDET
FAM134B$^{444-470}$ WT	*E. coli*	GPEEDTDTEEGDDFELLDQSELDQIESELY
FAM134B$^{453-460}$ (Δhelix)	Synthesis	DDFELLDQY
FAM134B$^{444-470}$ F455A	*E. coli*	GPEEDTDTEEGDDAELLDQSELDQIESELY
FAM134B$^{444-470}$ L458A	*E. coli*	GPEEDTDTEEGDDFELADQSELDQIESELY
FAM134B$^{444-470}$ E456A E462A	*E. coli*	GPEEDTDTEEGDDFALLDQSALDQIESELY
SEC62$^{355-376}$ WT	*E. coli*	GPHSSGNGNDFEMITKEELEQQTDY
SEC62$^{361-368}$ (Δhelix)	Synthesis	NDFEMITKY
SEC62$^{355-376}$ F363A	*E. coli*	GPHSSGNGNDAEMITKEELEQQTDY
SEC62$^{355-376}$ I366A	*E. coli*	GPHSSGNGNDFEMATKEELEQQTDY
SEC62$^{355-376}$ E364A E370A	*E. coli*	GPHSSGNGNDFAMITKEALEQQTDY
RTN3$^{245-264}$	Synthesis	ESPFEVIIDKAAFDKEFKDSY
RTN3$^{245-253}$	Synthesis	YESPFEVIID

Supplementary Table 5. Data collection and refinement statistics

	Atg40$^{237\text{-}252}$-Atg8	FAM134B$^{450\text{-}468}$-GABARAP	Sec62$^{361\text{-}376}$-GABARAP	RTN3$^{245\text{-}264}$-GABARAP
**Data collection**				
Space group	$P\ 3_2\ 2\ 1$	$P\ 6_3$	$P\ 2_1\ 2_1\ 2_1$	$P\ 4_1\ 2_1\ 2$
Cell dimensions				
a, *b*, *c* (Å)	74.72, 74.72, 57.09	81.89, 81.89, 33.32	42.89, 42.89, 144.01	137.41, 137.41, 67.44
α, β, γ (°)	90, 90, 120	90, 90, 120	90, 90, 90	90, 90, 90
Resolution (Å)	37.36 – 2.231 (2.311 – 2.231)	40.95 – 1.404 (1.454 – 1.404)	41.10 – 1.999 (2.071 – 1.999)	45.43 – 2.149 (2.226 – 2.149)
R_{merge}	0.1406 (0.5954)	0.0741 (0.6313)	0.1638 (0.9200)	0.0723 (0.8381)
$I / \sigma I$	13.78 (2.44)	18.80 (2.68)	9.27 (2.02)	20.14 (2.43)
Completeness (%)	99.58 (97.91)	97.71 (82.68)	99.73 (98.34)	99.87 (99.28)
Redundancy	9.6 (7.3)	9.7 (6.5)	6.5 (5.8)	13.2 (13.4)
**Refinement**				
Resolution (Å)	37.36 – 2.231	40.95 – 1.404	41.10 – 1.999	45.43-2.149
No. reflections	9227	24601	18676	35698
R_{work} / R_{free}	0.2042 / 0.2199	0.1915 / 0.2061	0.1656 / 0.1836	0.2062 / 0.2299
No. atoms				
Protein	1076	1106	2172	3272
	21	6		30
Ligand/ion				
Water	42	105	291	96
B-factors				
Protein	45.99	28.92	30.61	72.17

	54.62	44.45		124.31
Ligand/ion				
Water	44.72	38.64	34.55	57.28
R.m.s. deviations				
Bond lengths (Å)	0.008	0.006	0.007	0.008
Bond angles (°)	1.29	0.79	0.91	1.18

*Number of xtals for each structure is one. *Values in parentheses are for highest-resolution shell.

Supplementary ImageJ Script 1

```
//Z-stack images of Atg1-mCherry and Atg40-GFP were named as "Atg1-mCherry.tif" and “Atg40-GFP.tif", respectively

//find Atg1-mCherry puncta and create a mask image
selectWindow("Atg1-mCherry.tif");
run("Find Maxima...", "noise=500 output=[Single Points] exclude");
run("Find Maxima...", "noise=0 output=[Point Selection] exclude");
getSelectionCoordinates(xpoints, ypoints);
  radius=2.5;
  for (XY=0; XY<lengthOf(xpoints); XY++) {
    makeOval(xpoints[XY]-radius+0.5, ypoints[XY]-radius+0.5, 2*radius, 2*radius);
   run("Fill", "slice");
  }
rename("Atg1-mCherry-mask");

//find Atg40-GFP puncta and create a mask image
selectWindow("Atg40-GFP.tif");
run("Find Maxima...", "noise=5000 output=[Single Points] exclude");
rename("Atg40-GFP-mask");

//extract Atg40-GFP puncta that colocalize with Atg1-mCherry puncta and count the number of these puncta
imageCalculator("Multiply create", "Atg1-mCherry-mask","Atg40-GFP-mask");
rename("merge");
run("Analyze Particles...", "display");
```

Supplementary ImageJ Script 2

```
//Z-stack images of Atg40-mCherry and GFP-HDEL/Yop1-mNeonGreen were named as "Atg40-mCherry.tif" and "GFP-mNG.tif", respectively

//find Atg40-mCherry puncta and store the ROIs
selectWindow("Atg40-mCherry.tif");
run("Select All");
run("Size...", "width=2880 height=2880 depth=1 constrain interpolation=Bilinear");
run("Find Maxima...", "noise=2500 output=[Single Points] exclude");
run("Find Maxima...", "noise=0 output=[Point Selection] exclude");
getSelectionCoordinates(xpoints, ypoints);
radius=3;
for (XY=0; XY<lengthOf(xpoints); XY++) {
   makeOval(xpoints[XY]-radius, ypoints[XY]-radius, 2*radius, 2*radius);
   run("Fill", "slice");
   }
run("Select All");
run("Multiply...", "value=255.000");
run("Analyze Particles...", "add");

//measure the intensities of GFP-HDEL/Yop1-mNeonGreen colocalized with Atg40-mCherry-positive autophagosomes
selectWindow("GFP-mNG.tif");
run("Select All");
run("Size...", "width=2880 height=2880 depth=1 constrain interpolation=Bilinear");
num = roiManager("count");
roiManager("Show All");
for (n=0; n<=num-1; n++){
    roiManager("Select", n);
    roiManager("Measure");
};
```

Supplementary ImageJ Script 3

```
//a Z-stack image of GFP-HDEL/Yop1-mNeonGreen was named as "GFP-mNG.tif"
//find the GFP-HDEL/Yop1-mNeonGreen-positive ER regions and create a mask image
selectWindow("GFP-mNG.tif");
run("Select All");
run("Size...", "width=2880 height=2880 depth=1 constrain interpolation=Bilinear");
run("Duplicate...", "title=GFP-mNG-mask");
setAutoThreshold("Moments dark");
run("Convert to Mask");
run("Create Selection");
roiManager("Add");

//measure the total intensity of GFP-HDEL/Yop1-mNeonGreen
selectWindow("GFP-mNG.tif");
roiManager("Measure");
```
