# Supplementary material for: Super-assembly of ER-phagy receptor Atg40 induces local ER remodeling at contacts with forming autophagosomal membranes
Source: Nat Commun. 2020 Jul 3;11:3306. doi: 10.1038/s41467-020-17163-y (PMC7335187; doi:10.1038/s41467-020-17163-y)
Supplement: Supplementary file 4 — Description of Additional Supplementary Files [file 41467_2020_17163_MOESM4_ESM.pdf]

Supplementary Movie 1 | Coalesce of mCherry-Atg8-CC^{tetramer}-SNAP-GST-40C liquid droplets.

10 μM mCherry-Atg8-CC^{tetramer} was mixed with 10 μM SNAP-GST-40C and observed under a confocal microscope.

Scale bars, 5 μm
