# Supplementary material for: Super-assembly of ER-phagy receptor Atg40 induces local ER remodeling at contacts with forming autophagosomal membranes
Source: Nat Commun. 2020 Jul 3;11:3306. doi: 10.1038/s41467-020-17163-y (PMC7335187; doi:10.1038/s41467-020-17163-y)

Fig. 1a

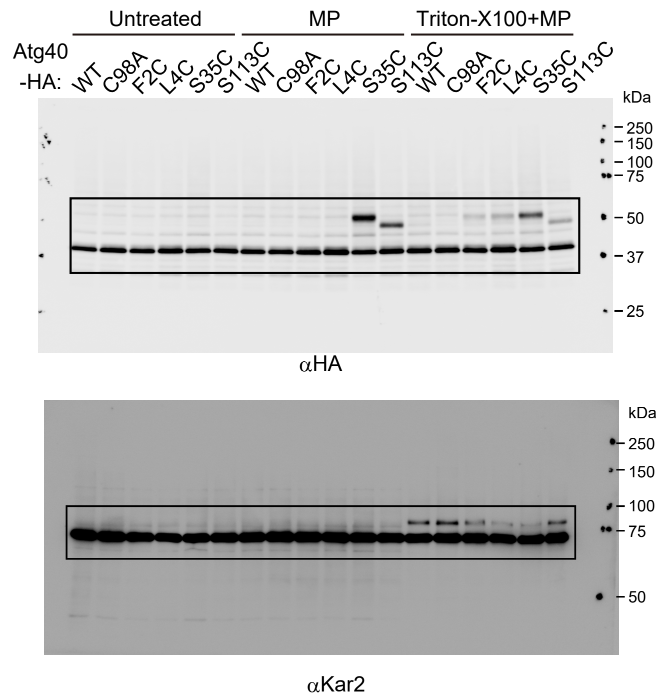

Fig. 2b

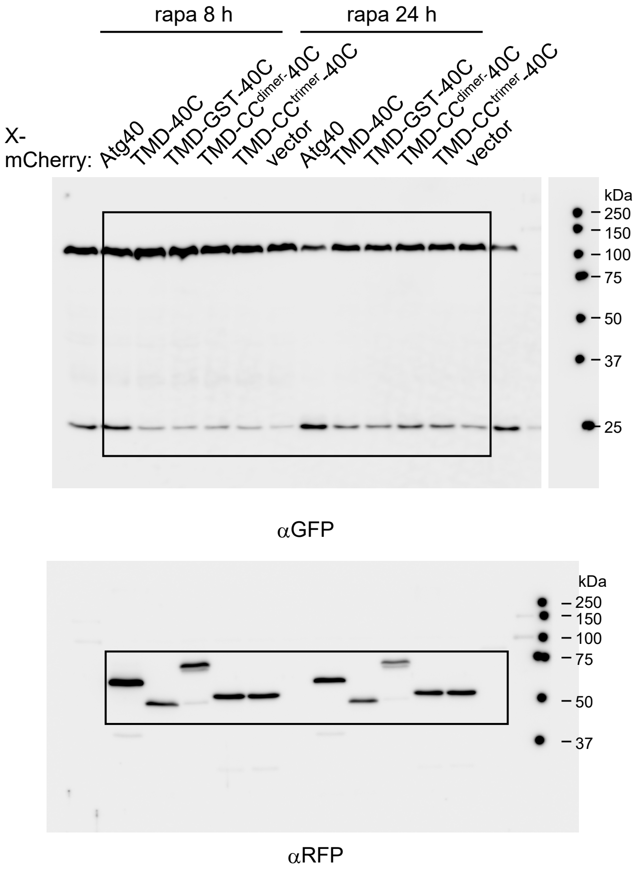

Fig. 2c

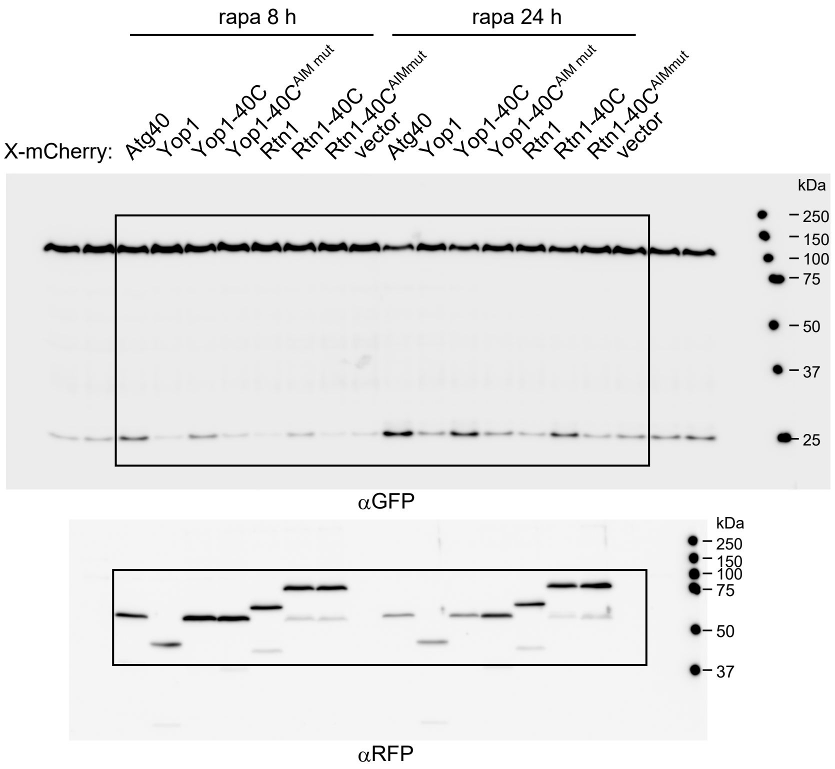

Fig. 4b

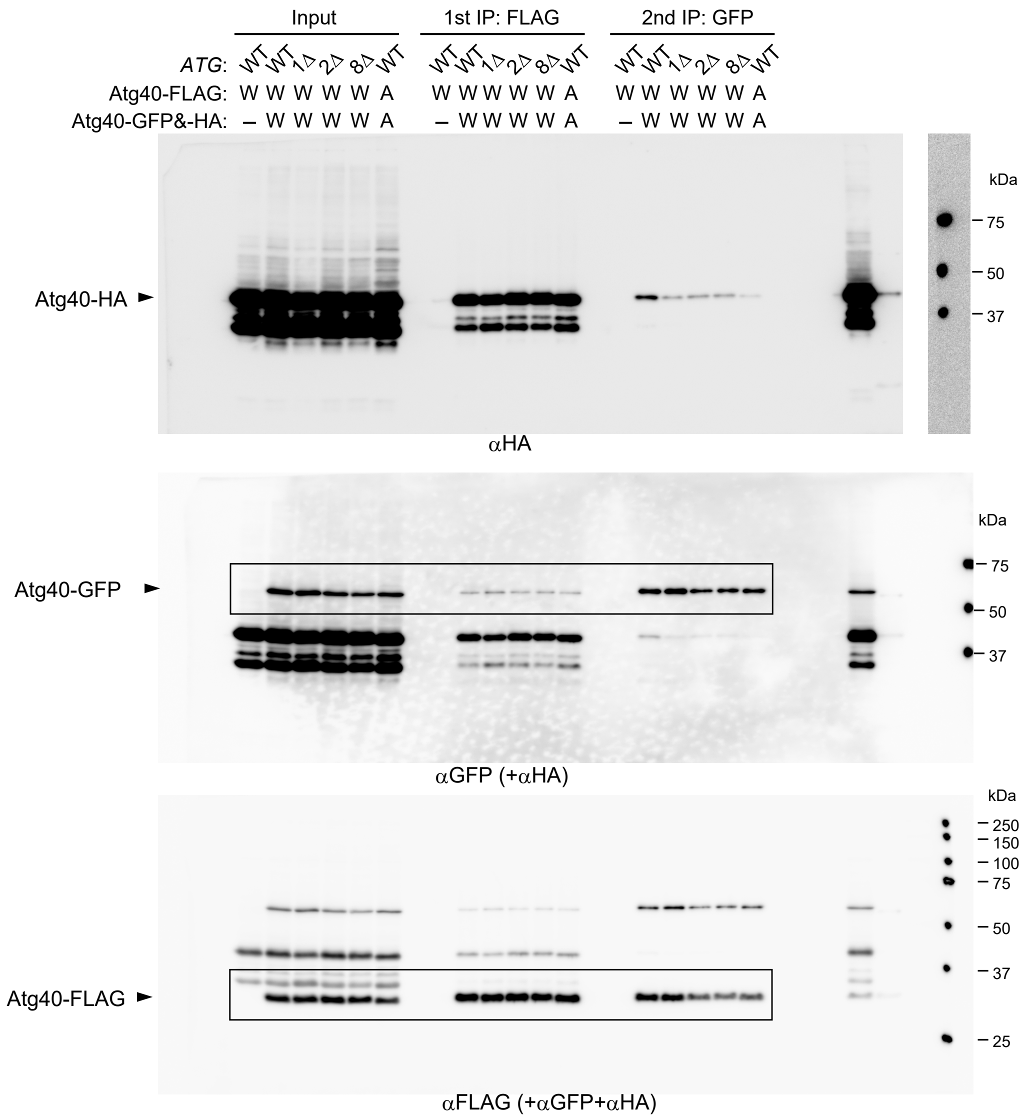
Input
1st IP: FLAG
2nd IP: GFP
ATG: WT WT 1Δ 2Δ 8Δ WT
Atg40-FLAG: W W W W W A
Atg40-GFP&-HA: – W W W W A
Atg40-HA
αHA
kDa
75
50
37
Atg40-GFP
αGFP (+αHA)
Atg40-FLAG
αFLAG (+αGFP+αHA)
250
150
100
25

Fig. 5c

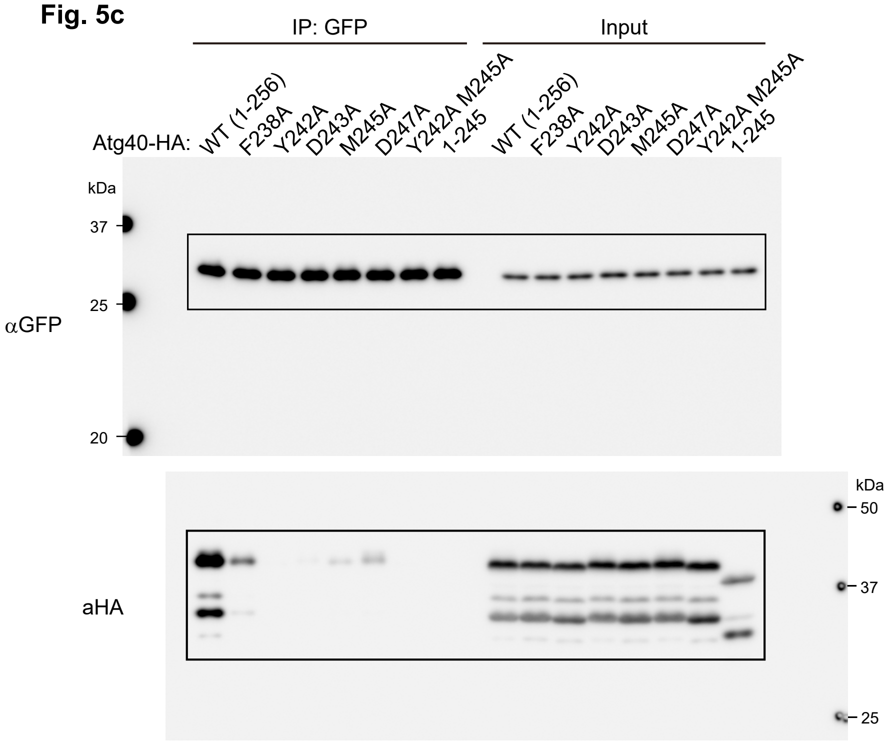
IP: GFP
Input
Atg40-HA: WT (1-256) F238A Y242A D243A M245A D247A Y242A M245A 1-245
kDa
37
25
20
αGFP
aHA
50

Supplementary Fig. 1a

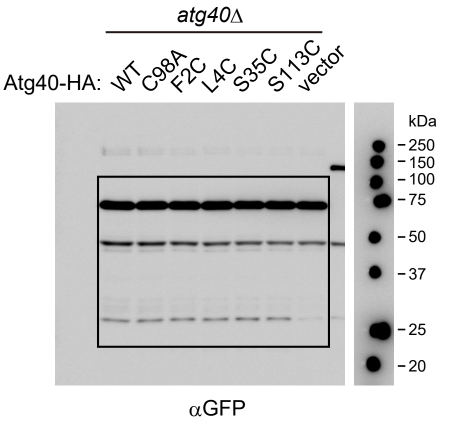

Supplementary Fig. 1d

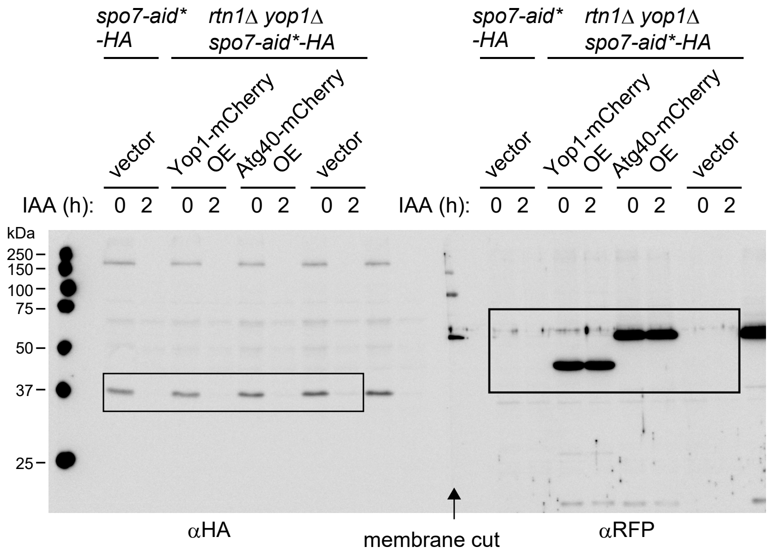

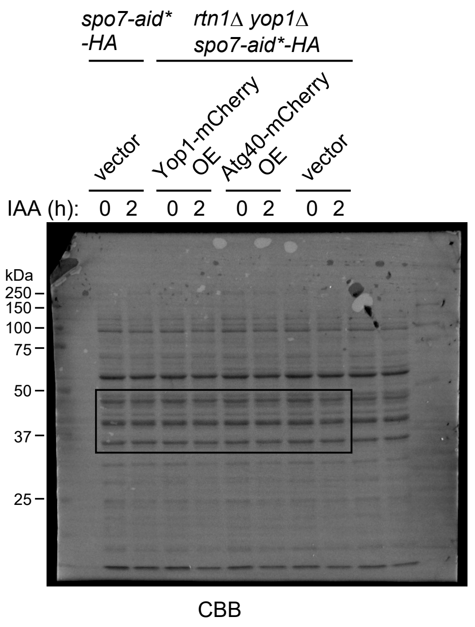

Supplementary Fig. 2a

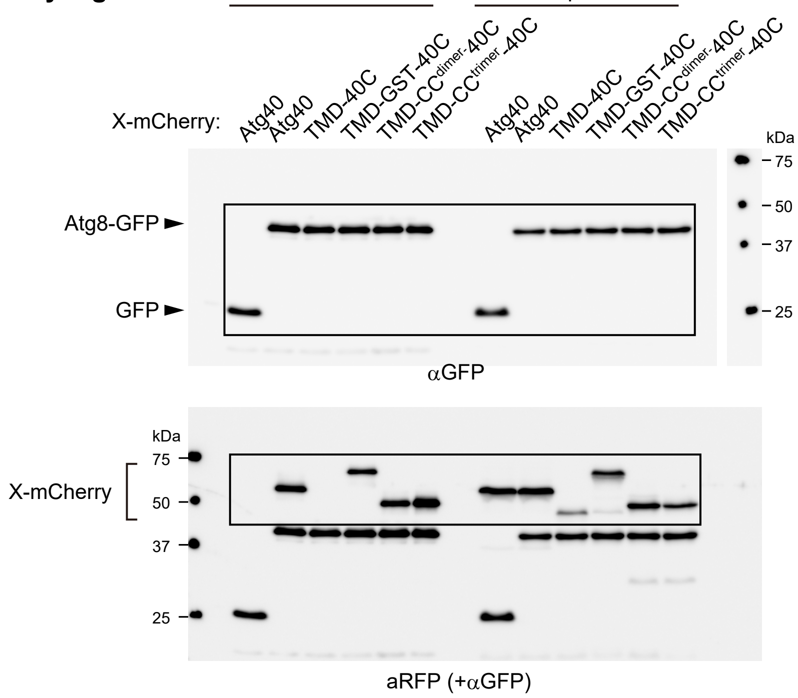

Supplementary Fig. 6c

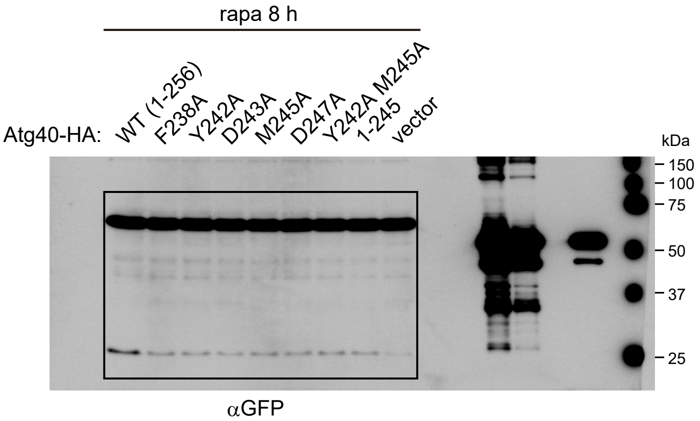

Supplementary Fig. 6e

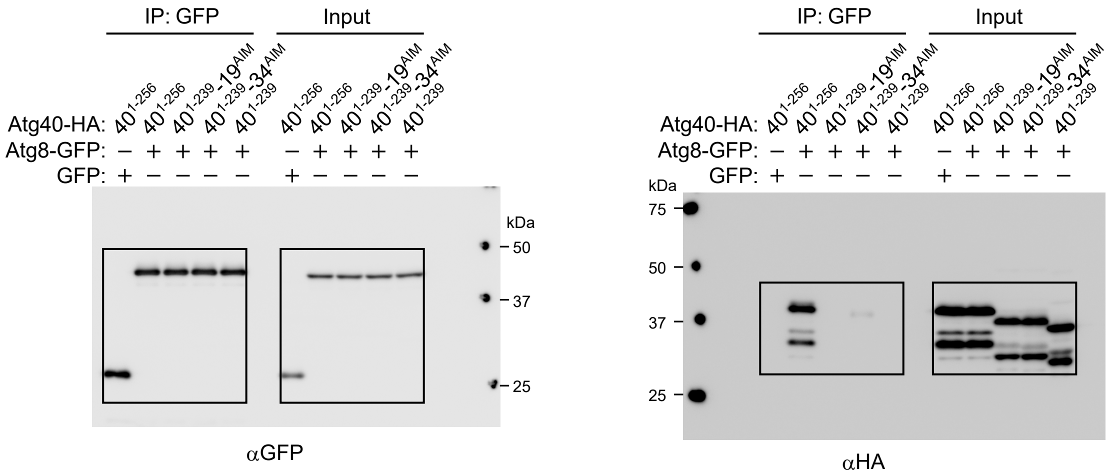

Supplementary Fig. 6f

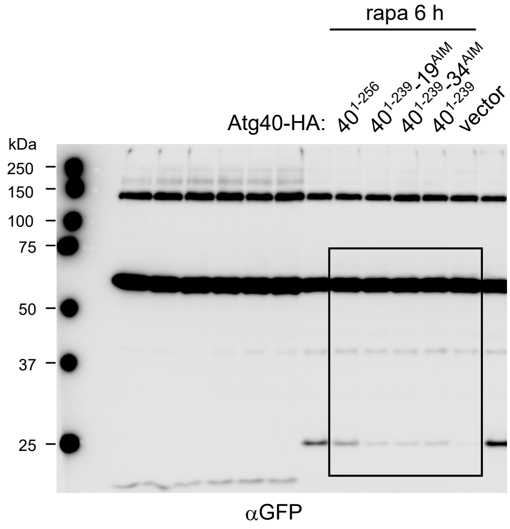

Supplement: Supplementary file 5 — Source data [file 41467_2020_17163_MOESM5_ESM.zip › Source_Data_for_blots.pdf]
